# Design Principles and Mechanistic Understandings of Non-Noble-Metal Bifunctional Electrocatalysts for Zinc–Air Batteries

**DOI:** 10.1007/s40820-024-01366-9

**Published:** 2024-03-26

**Authors:** Yunnan Gao, Ling Liu, Yi Jiang, Dexin Yu, Xiaomei Zheng, Jiayi Wang, Jingwei Liu, Dan Luo, Yongguang Zhang, Zhenjia Shi, Xin Wang, Ya-Ping Deng, Zhongwei Chen

**Affiliations:** 1 Power Battery and Systems Research Center, State Key Laboratory of Catalysis, Dalian Institute of Chemical Physics, Dalian Institute of Chemical Physics, Chinese Academy of Sciences, Dalian, 116023 People’s Republic of China; 2 Department of Chemical Engineering, University of Waterloo, Waterloo, ON N2L 3G1 Canada; 3 Institute of Carbon Neutrality, Zhejiang Wanli University, Ningbo, 315100 People’s Republic of China; 4 College of Materials and Chemistry, China Jiliang University, Hangzhou, 310018 People’s Republic of China

**Keywords:** Zinc–air batteries, Bifunctional electrocatalysts, Design principles, Mechanistic understandings

## Abstract

Zinc–air batteries (ZABs) are promising energy storage systems because of high theoretical energy density, safety, low cost, and abundance of zinc. However, the slow multi-step reaction of oxygen and heavy reliance on noble-metal catalysts hinder the practical applications of ZABs. Therefore, feasible and advanced non-noble-metal electrocatalysts for air cathodes need to be identified to promote the oxygen catalytic reaction. In this review, we initially introduced the advancement of ZABs in the past two decades and provided an overview of key developments in this field. Then, we discussed the working mechanism and the design of bifunctional electrocatalysts from the perspective of morphology design, crystal structure tuning, interface strategy, and atomic engineering. We also included theoretical studies, machine learning, and advanced characterization technologies to provide a comprehensive understanding of the structure-performance relationship of electrocatalysts and the reaction pathways of the oxygen redox reactions. Finally, we discussed the challenges and prospects related to designing advanced non-noble-metal bifunctional electrocatalysts for ZABs.

## Highlights

- The recent advances in non-noble-metal bifunctional electrocatalysts for zinc–air batteries are summarized with the design principles.
- The working mechanism are discussed to provide a comprehensive understanding of the structure-performance relationship of electrocatalysts and the reaction pathways of the oxygen redox reactions.
- The challenges and prospects related to designing advanced non-noble-metal bifunctional electrocatalysts for high performance zinc–air batteries are provided.

## Graphical abstract

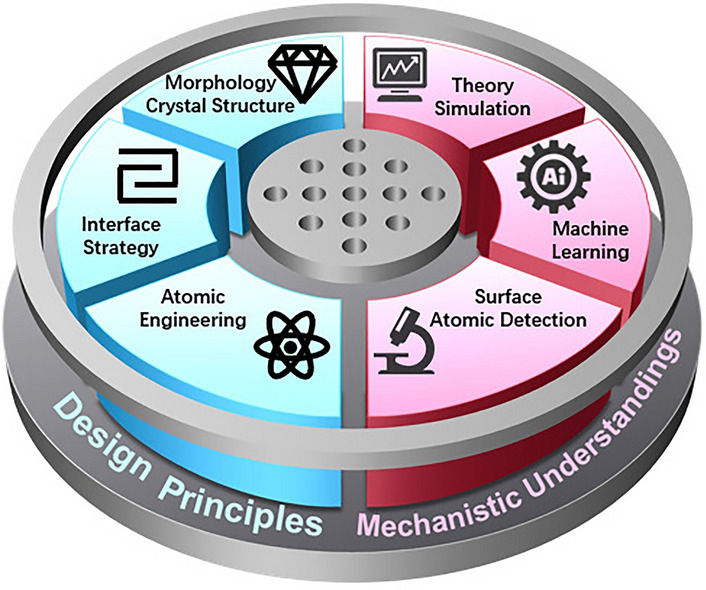

## Introduction

The consumption of large quantities of fossil energy accompanied by high carbon dioxide emissions has led to an energy crisis and climate change. Electrochemical energy storage and conversion are important for reducing the use of fossil energy and promoting a green, renewable, and efficient living environment. In the past decades, the field of battery systems has progressed considerably [1]. Three generations of batteries, including lead-acid battery [2], Ni-MH battery [3] and lithium-ion battery [4–6] (LIBs), were designed and commercialized. Among them, LIBs have received the most attention from researchers and have been popularized in various areas ranging from portable electric devices to large power systems, especially electric vehicles (EVs). However, the low specific energy density of the currently available LIBs greatly constrains the driving range of EVs. LIBs not only have inferior performance that cannot satisfy the growing demand of the industry, but the highly active Li also causes some serious safety issues [7–10]. To resolve these problems, many researchers have investigated next-generation battery systems that have a higher energy density and are safer and cheaper. Although several battery types were proposed as possible replacements, such as Na-ion [11], K-ion [12], Na–S [13], and Li–S battery [14], all of them had certain shortcomings that greatly limited their feasibility. For example, Na-ion batteries have a considerably lower energy density and greater risk compared to the available Li-ion batteries [15–17]. Similarly, for Li–S batteries, further studies are still required to address their low electronic conductivity due to the presence of sulfur, large variation in their volume, and severe shuttle effects during cycling [18, 19]. Therefore, it is challenging for these technologies to meet the increasing demands demand for battery systems in the future. Thus, high-energy/power density, durable, and safe energy storage systems need to be developed.

Metal-air batteries (MABs) have received much attention from researchers and have been extensively studied due to their high theoretical energy density (about 800–8100 Wh kg^−1^) that is several folds higher than that of LIBs (about 200–250 Wh kg^−1^) and renewable fuel resources, as they are powered by oxygen in the air [20]. Some researchers have investigated several MABs, including Li–O_2_ [21], Na–O_2_ [22], K–O_2_ [23], Zn–air [24], Mg–air [25], Al–air [26] and Fe–air [27] batteries. Typically, MABs are of two types based on the different types of electrolytes used. One type is based on alkali anodes and uses aprotic electrolytes, including Li, Na, and K, which are usually known as O_2_ batteries instead of MABs, as they use pure O_2_ as the cathode fuel to minimize the negative effects of CO_2_ and moisture on the anode [20]. The electrolyte used for Zn–air, Mg–air and Al–air is aqueous. Although researchers have used various strategies to make them practically feasible, three major concerns hinder their large-scale commercial application: (1) serious safety concerns regarding the high reactivity of these alkali anodes and flammable organic electrolytes; (2) high cost to meet the strict manufacturing criteria; (3) environmental pollution, such as metal/heavy metal-related, electrolyte contamination, air pollution, etc. [28, 29]. In contrast to alkali MABs, aqueous-based MABs have considerable advantages, including greater safety, decent conductivity, lower cost, and more eco-friendly. Thus, aqueous-based MABs have received much attention from researchers for further improving their electrochemical performance [30].

Although Mg–air and Al–air batteries have a very high theoretical energy density and discharge voltage, their poor rechargeability limits their commercial application. The problem occurs mainly because the electroreduction of Mg^2+^ and Al^3+^ in aqueous electrolyte is not feasible. Limitations in such primary battery configurations greatly decrease the practical values of the two batteries [31]. As Fe–air batteries are considerably cheaper than other systems, some rechargeable prototypes were developed, which showed cyclability of over 1000 cycles. However, the performance of iron electrodes is limited by the evolution of hydrogen and electrode passivation [32]. The company called Form Energy may solve the problems based on the "reversible oxidation of iron". The Fe–air batteries developed by Form Energy might scale up from a lab-scale prototype to a grid-scale plant, to provide 150 h of power at one-tenth of the cost of lithium batteries by 2025. Compared to the above-mentioned metal-air batteries (Table 1), Zn-air batteries (ZABs) are better MABs with a suitable theoretical energy density (1086 Wh kg^−1^), non-toxicity, low cost (3.00 $ per kg Zn), and safety.

**Table 1:** Comparison of various metal air batteries

Battery type	Cell voltage (V)	Specific energy (Wh kg^−1^)	Cost ($ per kg)	Electrolyte	Rechargeable	Advantage	Disadvantage
Li–O_2_	2.96	5930	185.1	Aprotic	Yes	High energy density	Unsafe operation, poor reversibility, poor cycle life
Na–O_2_	2.3	1680	2.7	Aprotic	Yes		
K–O_2_	2.37	1190	22.6	Aprotic	Yes		
Zn–air	1.65	1220	2.9	Aqueous	Yes	Suitable theoretical energy density, nontoxicity, low cost, safety	Lack of active and durable bifunction-al electrocatalysts on the cathode
Mg–air	3.09	5240	3.0	Aqueous	No	High theoretical energy density and discharge voltage	Poor rechargeability and practical values
Al–air	2.71	5780	2.6	Aqueous	No		
Fe–air	1.28	1080	0.5	Aqueous	Yes	Low cost, good cyclability	Evolution of hydrogen and electrode passivation

So far, some companies, such as EOS Energy Storage, Fluidic Energy, and ZincNyx Energy Solutions, have joined the investigation and contributed immensely to the research on ZABs, whereas the products are still at an early stage. Some limitations of anodes need to be addressed, such as dendrite formation, shape change, passivation, corrosion, and side reactions with electrolytes (hydrogen evolution reaction) during cycling [33]. Many studies have investigated ways to enhance the cycling lifetime by incorporating Zn, using strategies primarily involving the introduction of additives to the anode, electrolyte, or separator to resolve various problems associated with the anode [34–36]. Oxygen electrocatalytic redox reactions, including oxygen reduction reactions (ORR) for discharging and oxygen evolution reactions (OER) for charging, are the cathode reactions that occur at the air electrode. The key barrier lies on the side of the cathode, i.e., the lack of active and durable bifunctional electrocatalysts, which has motivated many researchers to use various techniques to identify suitable candidates [37].

The benchmark electrocatalysts are mostly made from precious metal materials, i.e., Pt/C for ORR and RuO_2_/IrO_2_ for OER; however, their high cost and low abundance limit their practical application [38, 39]. Therefore, studies on efficient and cheap bifunctional electrocatalysts are necessary to promote further commercialization of ZABs, although the task is challenging. The non-noble-metal electrocatalysts, including transition metal compounds, carbon-supported transition metal materials, and metal-free carbon, exhibit promising electrolytic performance.

Some previous reviews have discussed rechargeable ZAB technology for each part to the configuration of the whole battery [40–42], nonetheless, few studies have explained the state-of-the-art design strategies of non-noble-metal bifunctional electrocatalysts for ZABs concerning structure-performance relationships. To establish a positive paradigm, here, we presented a review emphasizing the recent progress in bifunctional electrocatalysts for application in rechargeable ZABs. We also provided detailed information and guidance for future design and development. First, we discussed the progress of ZABs in the past two decades and provided an overview of the key developments in this field. Then, we highlighted the working principles and the design of the electrocatalysts based on morphology, crystal structure, interface, and atomic level. We combined theoretical studies and advanced/in situ characterization technologies to discuss the structure–function relationship of electrocatalysts and the reaction pathways of the oxygen redox reactions. Finally, we discussed the challenges to the development of bifunctional electrocatalysts and summarized research prospects.

## History of Zinc–Air Batteries

Research on Zinc–air batteries started in 1866 when Georges-Lionel Leclanché developed the first ZABs using ammonium chloride solution [43]. Afterwards, Heise and Schumacher used an alkaline electrolyte, which exhibited a long duration of 12–18 months [44]. Although a high energy density of 200 Wh kg^−1^ for ZABs by virtue of the stack cell configuration, ZABs developed in a slow rate till the 1980s due to the increased research attention on H_2_–O_2_ fuel cells with advanced gas-diffusion electrodes. Then, the emergence of Li-ion batteries halted further research on ZABs. However, Li-ion batteries have limitations such as a low theoretical energy density, severe safety hazards caused by organic electrolytes, harmful to the environment, and scarcity of resources [45]. Hence, the application of ZABs using an aqueous electrolyte was proposed again because of their advantages of safety and environmental friendliness. The iteration of advanced techniques and theoretical computation modeling were combined to develop a more efficient way to design ZABs. One promising direction of research involves the development of electrocatalysts that can facilitate ORR and OER, which are critical processes in many electrochemical energy devices. In 2012, Chen et al. published a study in which they fabricated a bifunctional electrocatalyst that could simultaneously catalyze the ORR and OER [46]. Their seminal study provided new insights into the catalyst design of cathode reactions. In 2013, Dai et al. developed a new electrocatalyst with a high energy density (700 W h kg^−1^) [47]. After the development of bifunctional electrocatalysts for ORR and OER by Chen et al. and Dai et al., significant advancements were made in the design of efficient and durable catalysts for application in energy conversion and storage. Researchers have developed various strategies for improving the performance of bifunctional catalysts, including their morphology, structure, and atomic-level design. For example, Fu et al. implemented a strategy for developing bifunctional catalysts. They designed a durable and efficient three-dimensional bifunctional air electrode by building a hair-like Co_3_O_4_ nanowire array on a stainless steel mesh current collector [48]. The electrode developed exhibited superior performance for the ORR and OER; thus, they made a promising candidate for flexible energy storage applications. To understand the design principles of the catalyst for the cathode, Wu et al. used advanced characterization techniques and found that stable gas–solid-liquid triple-phase reaction areas synergistically optimized electron conduction, oxygen gas diffusion, and ion transport for electrocatalysis, which ultimately improved the performance of ZABs [49]. In 2021, Sun et al. used theoretical methods, such as density functional theory (DFT), to identify reaction mechanisms and processes at the molecular level. They designed an air cathode with a water-poor and zinc ion-rich inner Helmholtz layer, which facilitated stable operations in ambient air and good reversibility [50]. By systematically combining theory, experiment, characterization, and electrolyzer design, a new paradigm emerged, which promoted the practical application of ZABs. The development of zinc batteries as power batteries for electric vehicles can effectively reduce carbon emissions and further advance the transition to a future with cleaner energy. However, several challenges need to be addressed before zinc batteries can be used extensively for commercial purposes. These challenges include optimizing the performance of the battery, improving its durability, and reducing the cost of production to make it more commercially viable. Researchers have made significant progress in these areas, and thus, zinc batteries might play an important role in sustainable transportation in the future.

## Working Principles of the ZABs

### Battery Reaction Mechanisms

There are several key components, including an aqueous electrolyte, a separator, a Zn anode, and an air cathode. In most cases, ZABs use a concentrated alkaline solution containing Zn salts, usually ZnO or Zn(CH_3_COO)_2_, to maintain the electrolyte with high oxygen diffusion coefficients and ionic conductivity at about 640 mS cm^−1^ (30 wt% KOH solution at 25 °C). For the Zn anode, metallic plates or powders are mostly applied and undergo a two-electron redox of Zn^2+^/Zn couple during the discharge process (Eqs. 1 and 2). In contrast, Zn(OH)_4_^2–^ is reduced to metallic Zn by accepting electrons when charged [51].

1 $$ {\text{Zn}} + 4{\text{OH}}^{-} \leftrightarrow {\text{Zn}}\left( {{\text{OH}}} \right)_{4}^{2 - } + 2{\text{e}}^{-} $$

2 $$ {\text{Zn}}\left( {{\text{OH}}} \right)_{{4}}^{{{2} - }} \leftrightarrow {\text{ZnO}} + {\text{2OH}}^{-} + {\text{H}}_{{2}} {\text{O}} $$

Electrocatalysts associated with air electrodes simultaneously catalyze ORR and OER during the operation of ZABs. The ORR branch is usually illustrated as the following procedure: the catalyst first contacts with the O_2_ in the environment and adsorbs it at the active site, then the O–O bonds are broken, leading to the formation of oxygen intermediates, and finally, the produced OH^−^ diffuses into the electrolyte [52]. These reaction steps are shown below (* represents the active site of the catalyst):

Bidentate O_2_ adsorption:

3 $$ {\text{O}}_{{2}} + {\text{2H}}_{{2}} {\text{O}} + {\text{4e}}^{-} \to {\text{4OH}}^{-} $$

4 $${*}+{\text{O}}_{{2}} \to {\text{O}}_{2}^{*} $$

5 $$ {\text{O}}_{2}^{*} + {\text{H}}_{{2}} {\text{O}} + {\text{e}}^{-} \to {\text{HOO}}^{*} + {\text{OH}}^{-} $$

6 $$ {\text{HOO}}^{*} + {\text{e}}^{-} \to {\text{O}}^{*} + {\text{OH}}^{-} $$

7 $$ {\text{O}}^{*} + {\text{e}}^{-} + {\text{H}}_{{2}} {\text{O}} \to {\text{OH}}^{-} + {\text{OH}} $$

8 $$ {\text{OH}}^{*} + {\text{e}}^{-} \to {\text{OH}}^{-} +^{*} $$

End-on O_2_ adsorption:

9 $$ {\text{O}}_{{2}} + {\text{H}}_{{2}} {\text{O}} + {\text{2e}}^{-} \to {\text{HO}}_{{2}}^{-} + {\text{OH}}^{-} $$

10 $${*}+ {\text{O}}_{{2}} \to {\text{O}}_{2}^{*} $$

11 $$ {\text{O}}_{2}^{*} \to {\text{2O}}^{*} $$

12 $$ {\text{2O}}^{*} + {\text{2H}}_{{2}} {\text{O}} + {\text{2e}}^{-} \to {\text{2HO}}^{*} + {\text{2OH}}^{-} $$

13 $$ {\text{2HO}}^{*} + {\text{2e}}^{-} \to {\text{2OH}}^{-} +^{*} $$

An ORR may proceed along either a 4e^−^ route according to Eq. (3) or a 2e^−^ route as shown in Eq. (9). The specific reaction pathway depends on the configuration of the active sites in electrocatalysts. The adsorption of O_2_ can occur in two ways: bidentate O_2_ adsorption with two oxygen atoms coordinated with the electrocatalyst, and end-on O_2_ adsorption, i.e., one oxygen atom coordinated perpendicularly to the catalyst. The former adsorption method primarily occurs via the 4e^−^ route with direct hydroxyl formation (as shown in Eqs. 4–8), while the latter one usually leads to a 2e^−^ route with peroxide production (as shown in Eqs. 10–13). The peroxides formed in the 2e^−^ route is corrosive, which can destroy the cycling stability. In practice, those electrocatalysts which can direct the ORR toward the 4e^−^ route are in high demands [53].

The OER is the reverse of ORR but with important differences in the design of the catalyst. Considering that the OER process has decent activity and stability, metal-based materials are used to illustrate the OER mechanism. The OER process in an alkaline electrolyte is shown in the equations below, where M represents the active site in the electrocatalysts and “ads” represents the adsorbed species at the active sites. The OER pathway begins with the coordination of hydroxyl ions at the active sites, as shown in Eq. (14), and then, several steps occur according to two routes. In one route (Eqs. 15 and 16), O_2_ is produced by the coupling reaction between the two M–O_ads_ intermediates. Another route involves Eqs. (15)–(17) and (18), where M–OOH_ads_ is generated by reactions between M–O_ads_ and OH. Then, M–OOH_ads_ further reacts with OH^−^ to form O_2_. Theoretically, the reaction barrier of the former reaction route is higher [54].

14 $$ {\text{M}} + {\text{OH}}^{-} \to {\text{M}} - {\text{OH}}_{{{\text{ads}}}} + {\text{e}}^{-} $$

15 $$ {\text{M}} - {\text{OH}}_{{{\text{ads}}}} + {\text{OH}}^{-} \to {\text{M}} - {\text{O}}_{{{\text{ads}}}} + {\text{H}}_{{2}} {\text{O}} + {\text{e}}^{-} $$

16 $$ {\text{M}} - {\text{O}}_{{{\text{ads}}}} + {\text{M}} - {\text{O}}_{{{\text{ads}}}} \to {\text{2M}} + {\text{O}}_{{2}} $$

17 $$ {\text{M}} - {\text{O}}_{{{\text{ads}}}} + {\text{OH}}^{-} \to {\text{M}} - {\text{OOH}}_{{{\text{ads}}}} + {\text{e}}^{-} $$

18 $$ {\text{M}} - {\text{OOH}}_{{{\text{ads}}}} + {\text{OH}}^{-} \to {\text{M}} + {\text{O}}_{{2}} + {\text{H}}_{{2}} {\text{O}} + {\text{e}}^{-} $$

Although alkaline electrolytes are commonly used in ZABs, they are susceptible to zinc dendrite formation, ZnO precipitation, electrode corrosion, and electrolyte degradation, which can decrease cycle stability and battery life. To overcome these challenges and improve the performance of ZABs, neutral or near-neutral electrolytes are used. In a neutral medium, the reactions at the air cathode are the same as those in alkaline electrolytes, but the reactions at the zinc anode are different due to a significant decrease in the concentration of Zn(OH)_4_^2−^ caused by the absence of OH^−^. This can be seen in Eqs. (19–21).

19 $$ {\text{Air}}\;{\text{cathode}}{:}{\text{O}}_{{2}} + {\text{2H}}_{{2}} {\text{O}} + {\text{4e}}^{ - } \to {\text{4OH}}^{ - } $$

20 $$ {\text{Zinc}}\;{\text{anode:}}\;{\text{Zn}}^{{{2} + }} + {\text{2e}}^{ - } \to {\text{Zn}} $$

21 $$ {\text{Total}}\;{\text{reaction}}{:}{\text{ 2Zn}} + {\text{O}}_{{2}} \to {\text{2ZnO}} $$

Many types of electrolytes have been developed, whereas alkaline electrolytes are the most commonly used because the catalysts in them are more stable and have higher activity [55]. As many studies are already available on electrolytes [56, 57], we have not discussed them in this study.

### Battery Performance Evaluation

**Fig. 1:**
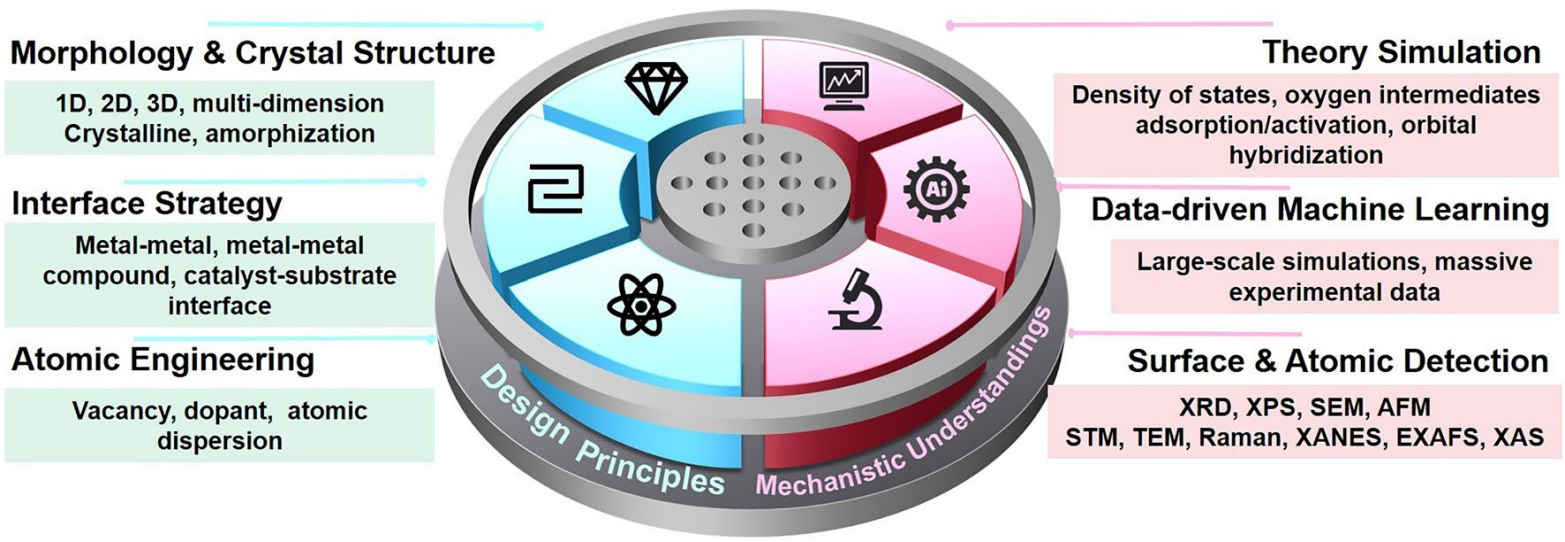
The scope of this review, which covers three aspects in the sequence of catalysts and analytical engineering

**Fig. 2:**
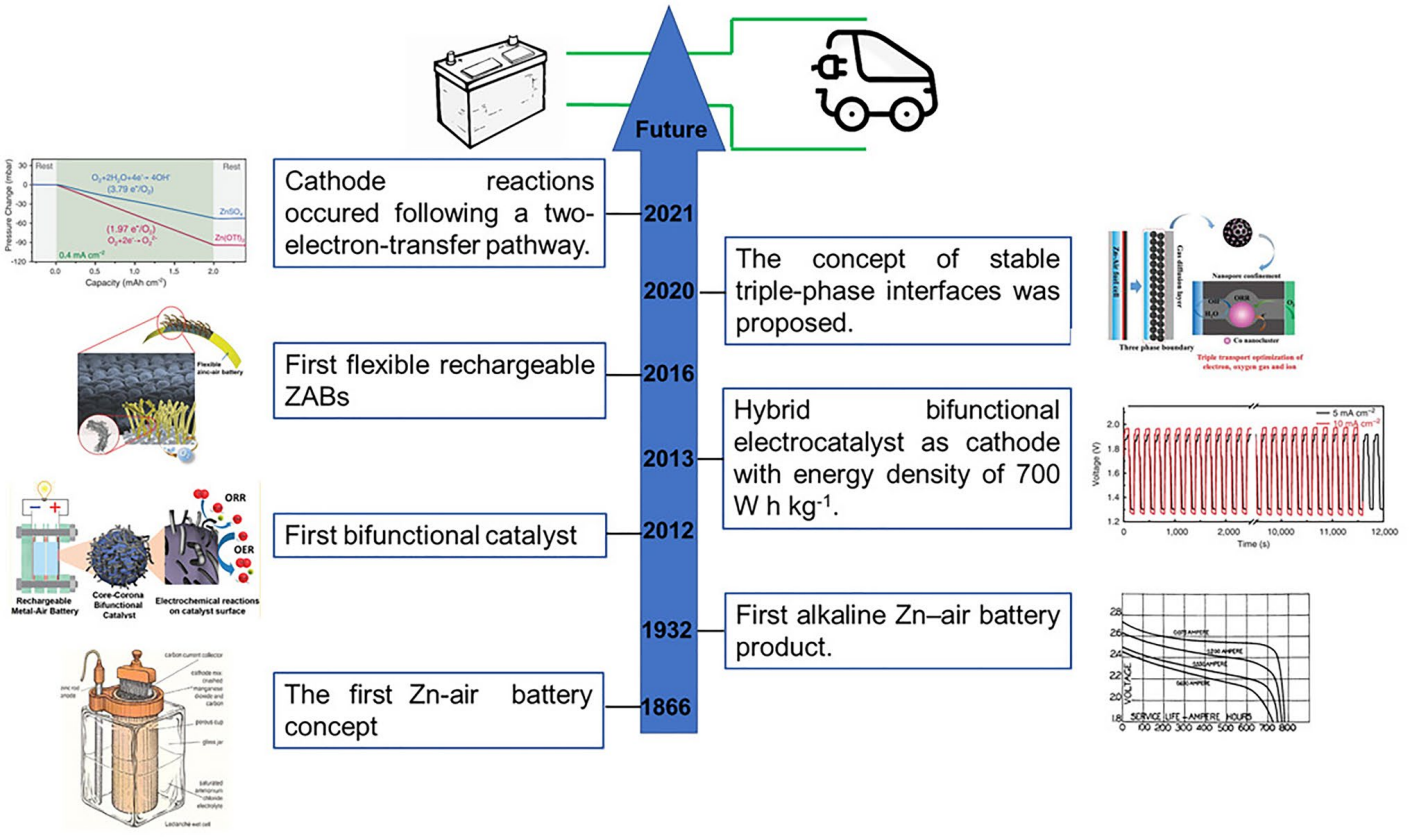
Schematic illustration of the progress of zinc–air batteries. Reprinted with permission from Ref. [43, 44, 46–50]

**Fig. 3:**
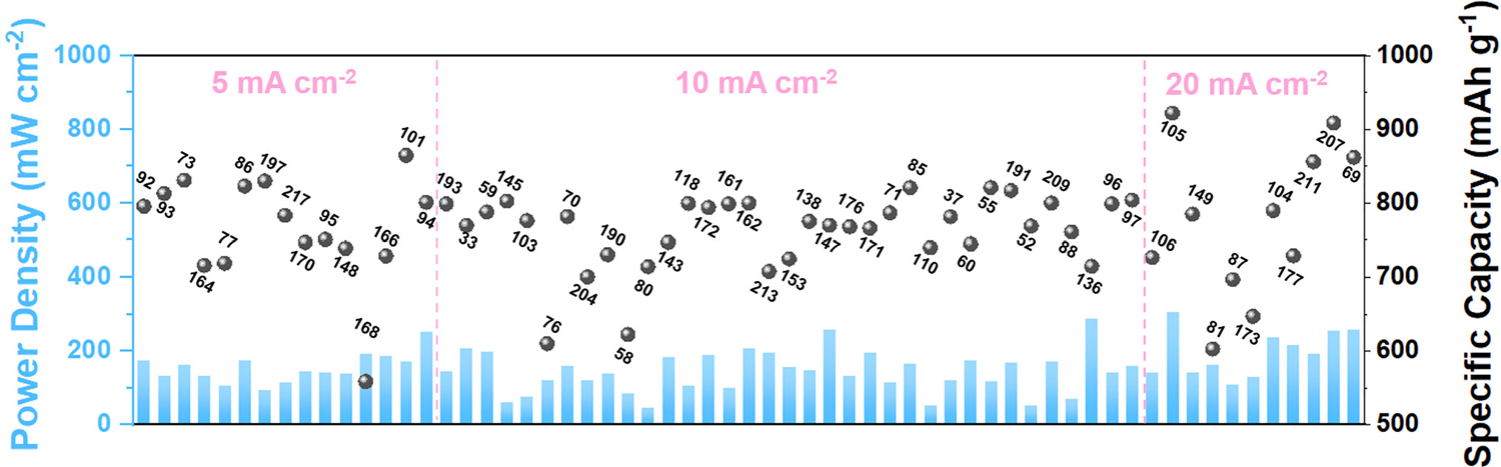
The distribution of the performance of the reported bifunctional electrocatalysts in recent three years

The performance of ZABs is generally measured using two parameters: activity and rechargeability. The activity of ZABs is assessed by discharge–charge voltage polarization and power density, which are influenced by the bifunctionality of the electrocatalyst [37, 58]. Rechargeability (or cycle ability) reflects the stability and durability of bifunctional electrocatalysts and is determined by the lifetime of ZABs in cycling tests at various current densities [59, 60]. The growth of the anode dendrite and electrolyte loss or leakage greatly constrains the cycling lifetime. The cathode is the most expensive part of the battery, and it strongly influences the performance and longevity of the battery. In the following section, we have discussed bifunctional electrocatalysts at the cathode (Figs. 1, 2 and 3).

## Design Principles for Bifunctional Electrocatalysts in ZABs

The benchmark electrocatalysts are mostly made of precious metal materials, i.e., Pt/C for ORR and RuO_2_/IrO_2_ for OER. However, these noble-metal materials are expensive, scarce, and do not exhibit high catalytic performance for ORR and OER. Therefore, research on efficient and cheap bifunctional electrocatalysts is necessary (although challenging) for commercializing ZABs. In this section, the contemporary bifunctional electrocatalysts are divided into three major categories, including carbon materials, metal compounds, and metal/carbon hybrids. To provide an overview of this field and drive future research, a thorough and methodical recapitulation of bifunctional capabilities is necessary. Data on catalysts has been collected and statistically analyzed in recent years to determine the distribution of electrocatalytic performance and guide the rational design of future electrocatalysts. All summarized data are presented in Fig. 3, which shows the electrochemical performance restricted within a power density and specific capacity of 300 mW cm^−2^ and 950 mAh g^−1^, respectively. Although significant progress has been made, the performance of ZABs, especially the activity of the ORR and OER at the cathode, is still challenging.

Oxygen electrocatalysis occurs at the triple-phase region, where the electrocatalyst contacts the electrolyte and O_2_. To develop an efficient bifunctional electrocatalyst, two general design principles are mainly considered at the triple-phase boundary. The first one involves increasing the number of active sites on the surface, which is usually achieved by increasing the catalytic surface area or optimizing the porosity of electrocatalysts. The other one involves altering the configuration of the active sites and manipulating the electrocatalytic efficiency of single sites. The ideal non-noble-metal bifunctional electrocatalysts for ZABs should be highly porous, inexpensive, eco-friendly, and have an optimal bond strength between zinc cations and oxygen/hydroxide species, small steric hindrance during charge transfer, superior conductivity, excellent chemical, and structural stability in the electrolyte, and a high surface area.

To summarize, recent advancements made in the design principles for ZAB cathode electrocatalysts fall under four main categories: (1) morphology design, which encompasses aspects such as shape, size, facet, porous size, and surface roughness; (2) crystal structure tuning, which pertains to crystalline and amorphous types; (3) interface strategy, which is beneficial to create or improve the catalytic activity, even accelerate the mass diffusion/charge during the electro-catalytic process; (4) atomic engineering, which involves techniques such as creating vacancies and dopants, as well as, atomic dispersion. These four methods can also interact and compound with each other. For example, shape and defects can both increase the surface area, which can increase the number of active sites. Facets and dopants can improve conductivity and enhance the stability of ZABs.

### Morphology Design

Geometrical merits attribute the superior performance of ZABs to the high specific surface area, beneficial architecture, and secondary structure. Specifically, the high surface area provides abundant electrocatalytic sites, and the pore structure favors electrolyte penetration. The secondary or hierarchical morphology can contribute to preserving the structural integrity and, therefore, to maintaining high electrocatalytic stability [61–65]. The different nanostructures can facilitate advancements in various ways. Based on previous geometrical assessments, a wide range of structures was obtained, including zero-dimensional nanoparticles, one-dimensional (1D) nanorods/nanofibers/nanotubes, two-dimensional (2D) nanosheets/nanoplates, and three-dimensional (3D) hierarchical structures. A multidimensional composition is also a common approach to morphology design.

#### Zero-dimension

**Fig. 4:**
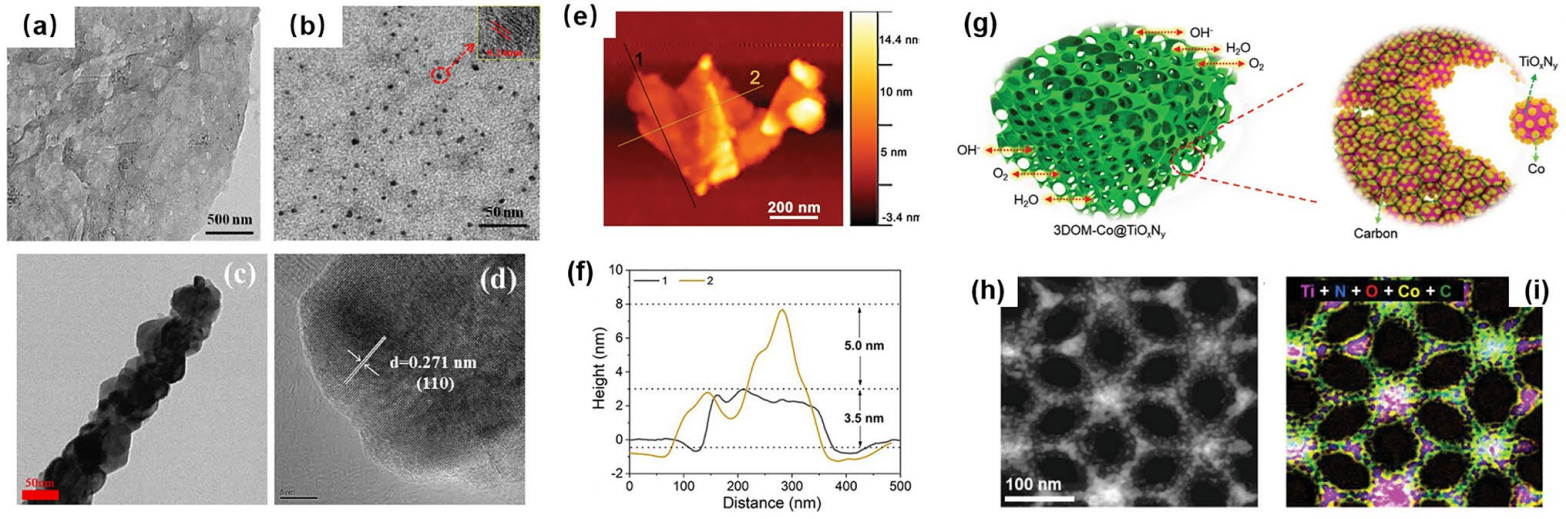
**a** TEM and **b** HRTEM images of the graphene quantum dots. Reprinted with permission from Ref. [68]. **c** TEM and **d** HRTEM of 1D La_0.8_Sr_0.2_Mn_0.9_5Co_0.05_O_3_ nanowires. Reprinted with permission from Ref. [75]. **e** AFM image and **f** the corresponding height profiles of CoO nanosheets. Reprinted with permission from Ref. [63]. **g** Schematic illustration and **h** STEM image, and **i** elemental mapping (Ti, Co, O, N, and C) of 3DOM titanium oxynitride. Reprinted with permission from Ref. [87]

Quantum dots (QDs), with sizes below 10 nm, have recently emerged as a pioneering class of nanomaterials, exemplifying the characteristics of 0D materials [66]. Among these, carbon quantum dots, ranging in diameter from 5 to 20 nm, have been extensively investigated for their role as metal-free electrocatalysts in ORR [67]. In a noteworthy contribution, Fan et al. demonstrated the synthesis of graphene quantum dots at low temperatures, employing a facile method that yielded a uniform nanometer-sized distribution, as visually depicted in Fig. 4a, b [68]. It's crucial to emphasize that quantum dots seldom exist independently; rather, they are typically integrated onto a carrier platform. A more comprehensive exploration of this phenomenon will be presented in the upcoming Multidimensional Composition Sect. 4.1.5. This section aims to delve deeper into the synergistic effects and multifaceted compositions that arise when quantum dots are coupled with specific carriers, providing a holistic understanding of their functional roles in various applications.

#### One-dimension

One-dimensional materials, encompassing nanofibers, nanowires, and nanotubes, serve as versatile building blocks with a multitude of applications [69], particularly in various energy storage-related contexts [70, 71]. Their distinctive electrical and optical properties, coupled with a high specific surface area, confer substantial advantages over traditional materials [72, 73]. In an insightful study, Li et al. successfully synthesized a one-dimensional hierarchical ternary nanocomposite of MnO@CNT@Co through a facile single-step calcination reaction. The porous 1D hierarchical nanostructure, in tandem with multiple active sites, collectively contributes to the exceptionally high bifunctional electrocatalytic performance and efficiency of MnO@CNT@Co in catalyzing both ORR and OER [74]. In a parallel effort, Li et al. employed a molten salt template method to synthesize one-dimensional La_0.8_Sr_0.2_Mn_0.95-_Co_0.05_O_3_ nanowires, as illustrated in Fig. 4c, d. The introduction of doped-Co in the nanowires resulted in the alteration of the band center of O 2*p*, Mn 3*d*, and Co 3*d*, effectively reducing the energy barriers of oxygenated intermediate species [75]. This strategic modification underscores the significance of tailoring the composition of one-dimensional materials to achieve optimized electrocatalytic performance in energy-related applications.

#### Two-dimension

Two-dimensional (2D) atomically thin catalysts are ideal platforms for ORR and OER [76, 77]. They have certain advantages, for example, (1) the charge distribution in the unique layer structure facilitates electron transfer; (2) the thin structure and strong in-plane chemical bonds improve the catalytic activity and offer superior mechanical strength; (3) the strong in-plane bonds with the thin structure offer superior mechanical strength; (4) the large aspect ratios (thin-layer) and the large number of exposed atoms on the surface provide a large specific surface area and a large number of active sites; (5) the 2D architecture provides a feasible and ideal model to modify its electronic distribution [78–82]. The CoO nanosheets fabricated by Tian et al. (Fig. 4e, f) were thin and had a high specific surface area and sufficient atomic utilization, leading to high rates of ORR/OER [63]. Many researchers use 2D MXenes as cathode materials in ZABs because they have high electrical conductivity, hydrophilicity, and mechanical strength. These properties facilitate efficient electronic charge transport at the electrode–electrolyte interface and promote fast Zn^2+^ diffusion, which greatly improves electrode kinetics, as proposed by Huang et al. [83].

#### Three-dimension

The development of three-dimensional (3D) physical structures has facilitated the emergence of new phenomena, such as restructuring and multiscale effects, which are attributed to the microstructure, dimensionality, and morphology of the materials [84–86]. Liu et al. synthesized 3D ordered microporous (3DOM) titanium oxynitride (Fig. 4g–h), in which the large diffusion channels for oxygen species and highly accessible active sites facilitated mass transfer; they were also quite durable [87]. Inspired by the former research, similar work by Wang et al. reported 3D ordered microporous P-doped Co_3_O_4_ (3DOM P-Co_3_O_4−*δ*_) [88]. Deng et al. found that the core–shell structure enhances the accessibility of active sites and widens the channels, which facilitates the diffusion of oxygen species. By encapsulating (Mg, Co)_3_O_4_ in N-doped graphitized carbon, the researchers recorded a highly efficient bifunctional oxygen electrocatalytic activity that outperformed even the most advanced noble-metal benchmarks [89]. Metal–organic frameworks (MOFs) with porous structure, high surface area and increased number of active sites, have attracted great interest of researchers [90]. Wang et al. designed porous cake-like ZnMn–NC by loading Mn into Zn–MOF and pyrolysis, resulting in an enhanced ORR activity [91]. The 3D electrocatalysts with many exposed active sites accelerated the activation of reactants/intermediates, and the irregular morphological features optimized the triple-phase interface [92, 93].

#### Multi-dimension

**Fig. 5:**
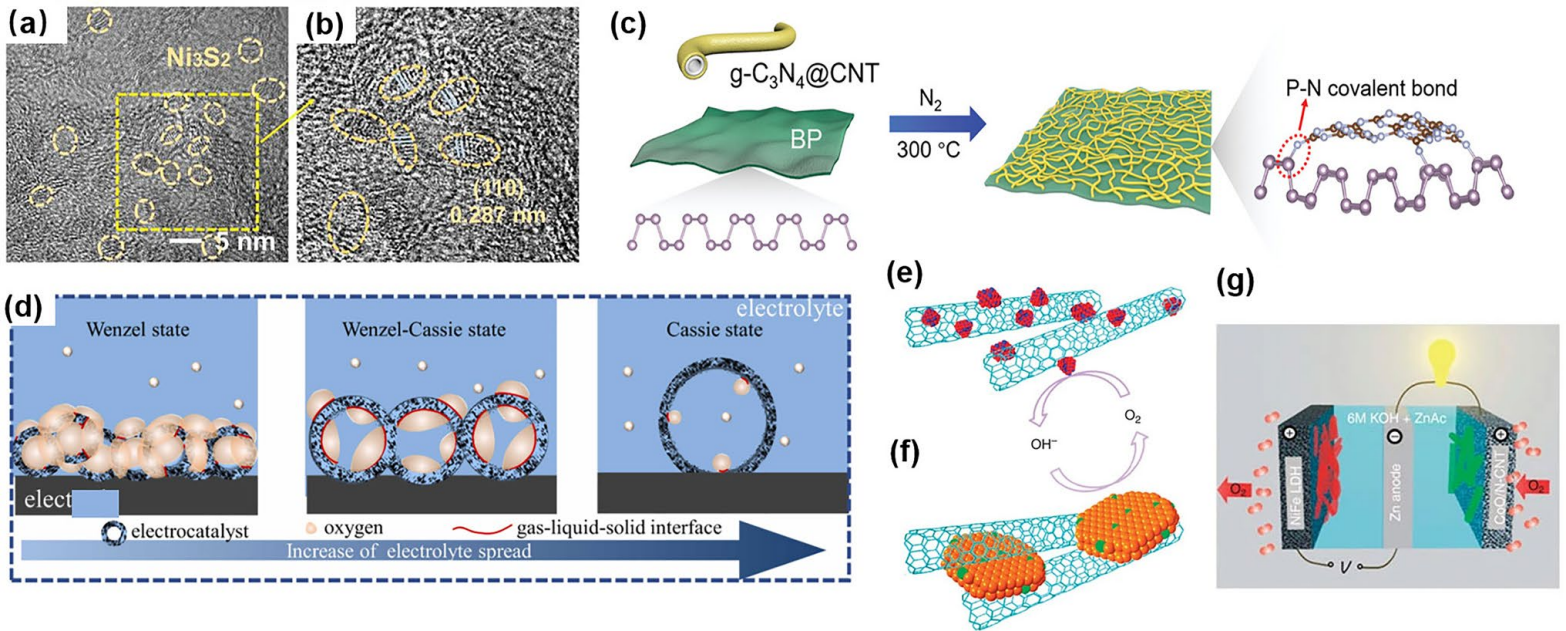
**a**, **b** HRTEM images of Ni_3_S_2_ quantum dots. Reprinted with permission from Ref. [100]. **c** Schematic illustration of 2D BP-CN-c. Reprinted with permission from Ref. [99]. **d** A diagrammatic representation of electrolyte diffusion state on hollow CoFe–NC–x. Reprinted with permission from Ref. [102]. **e**–**g** CoO and Ni–Fe-layered double hydroxide with CNT hybrids for the electrocatalysis of ORR and OER. Reprinted with permission from Ref. [47]

In multidimensional composite catalysts, different types of materials or structures are combined to create a composite structure with enhanced catalytic properties [94, 95]. Multidimensional composite catalysts are used extensively as they have higher catalytic activity, better selectivity, and improved stability than traditional catalysts [96, 97]. For example, Guo et al. introduced graphene quantum dots into Ni–Fe-layered double hydroxides as an air cathode for ZABs. The electrostatic adsorbing effect of graphene quantum dots improved the OER [98]. Another example was reported by Xu et al., who developed a facile hydrothermal and pyrolysis procedure to prepare Ni_3_S_2_ quantum dots on carbon nanosheets (Fig. 5a, b). By using this methodology, the robust bifunctionality of the air cathode exhibited ultra-long cyclability with a high discharge capacity, which probably resulted from the quantum size effects, oxygen deficiency, and a porous texture [99]. Wang et al. synthesized black phosphorus nanosheets with graphitic carbon nitride (denoted BP-CN-c) (Fig. 5c) with a mean thickness of 3.7 ± 1.3 nm using an electrochemical delamination method, which exposed the atoms and active lone pair electrons to promote the adsorption of OOH* [100]. One-dimensional structures provide more surface area and better transmission channels, while three-dimensional structures offer more reaction sites and greater stability. By combining these two structures, the catalytic efficiency and selectivity of a catalyst can be further improved, while reducing the toxicity and cost of the catalyst [101]. This can help in meeting the needs of different catalytic reactions. Some researchers synthesized a 3D nano-forest architecture composed of CoFe alloy and 1D N-doped CNTs grown on carbon nanofibers via electrospinning and two-stage heat-treatment [102]. The as-prepared CoFe@NC had a stable 3D structure, high conductivity, and hierarchical pores, which imparted high power density, low voltage polarization, and stable cycling compared to commercial ZABs (Fig. 5d). Zhuang et al. also reported similar findings; they described how the substantial surface curvature within mesoporous hollow spheres can produce a fortified air pocket and strengthen the air cushion when submerged underwater. Conversely, the reduced curvature resulting from an increased internal diameter can promote gradual electrolyte infiltration. Further experiments showed that the rapid entry and exit of reactants and products on an optimized triple-phase interface facilitated a low Tafel slope of ORR [103]. Dai et al. developed a hybrid oxygen reduction catalyst consisting of CoO and carbon nanotubes and an oxygen evolution catalyst made from Ni–Fe-layered double hydroxide and used them as cathodes (Fig. 5e, f). They conducted preliminary experiments on rechargeable Zn–air batteries using a tri-electrode setup. In this setup (Fig. 5g), OER and ORR electrocatalysts were applied to two separate electrodes for charging and discharging, respectively. The ORR electrode was connected only to the Zn anode for discharging, which prevented exposure to positive potentials. This configuration allowed for the decoupling of the two electrocatalysts, which enabled individual optimization [47].

By combining the advantages of different dimensional structures and materials, multidimensional composite catalysts can significantly enhance the catalytic activity and stability of ORR and OER [94, 104]. Additionally, the design and synthesis of multidimensional composite catalysts can be tailored for specific applications by controlling the composition, structure, and morphology of the catalysts [105, 106]. However, further studies are needed to elucidate the underlying mechanisms and optimize the performance of multidimensional composite catalysts for practical applications.

### Crystal Structure Tuning

#### Crystalline

**Fig. 6:**
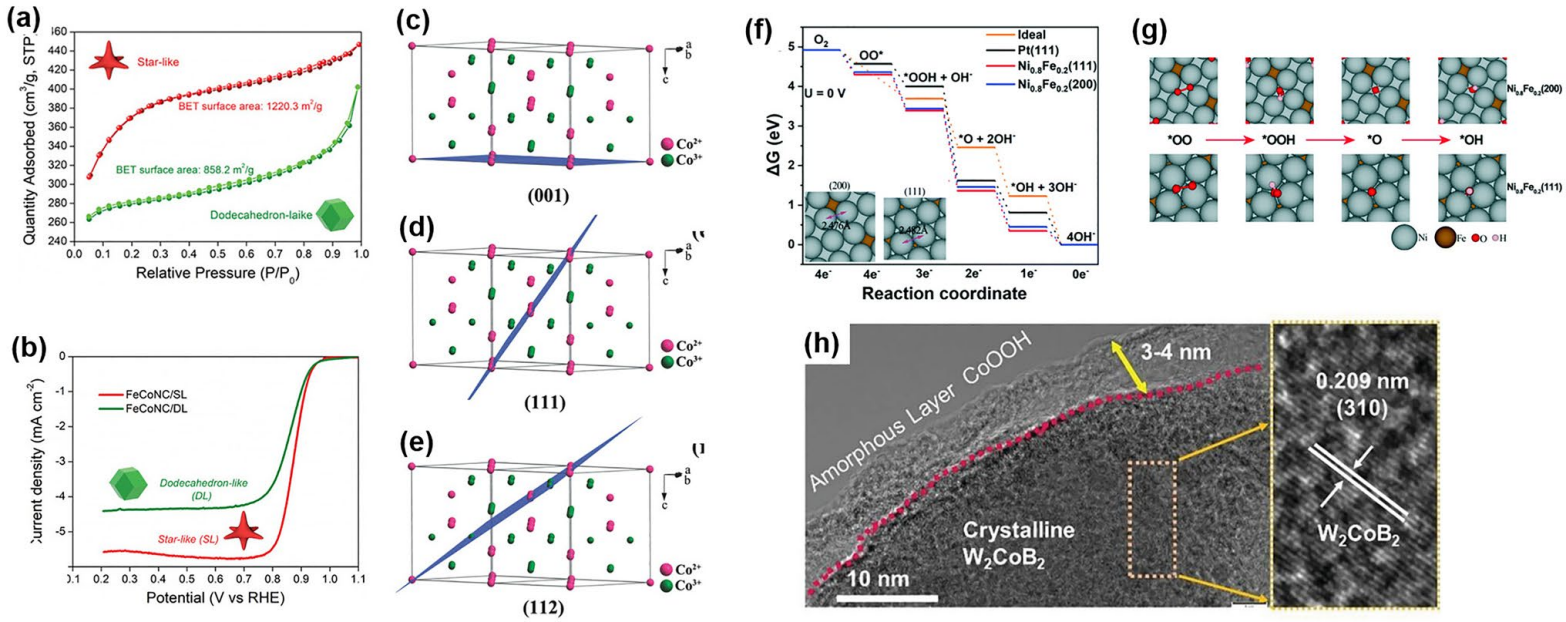
**a** The BET surface areas and **b** LSV curves of ORR of FeCoNC with star-like and dodecahedron-like structures. Reprinted with permission from Ref. [113]. **c**–**e** The Co^2+^/Co^3+^ surface atomic configurations and the corresponding side views of calculated O_2_-adsorption matter on different planes. Reprinted with permission from Ref. [116]. **f**, **g** Gibbs free energy and processes related to the ORR steps on the (111) and (200) facets of Ni_0.8_Fe_0.2_. Reprinted with permission from Ref. [117]. **h** TEM and HRTEM images of crystalline W_2_CoB_2_ with amorphous CoOOH layer. Reprinted with permission from Ref. [133]

The crystal structure of electrocatalysts strongly influences electrocatalytic processes, and the rational tuning of the surface structure to expose the active sites can enhance the performance of ZABs [107–110]. For example, Li et al. described a general method for synthesizing ultrafine Co-Mn spinel with cubic (c-spinel) and tetragonal (t-spinel) crystallographic symmetry. Compared to t-spinel, c-spinel contains more catalytic sites and a higher average oxidation state of Mn (> 3), which results in stronger binding of O_2_ molecules and improves electron conduction (via hopping) and charge transfer (through redox reactions), thus favoring electrocatalysis [111]. Lee et al. examined spinel-structured oxides (Co_3_O_4_, (Ni, Co)_3_O_4_, and (Mn, Co)_3_O_4_), which exhibited different levels of catalytic activity for both ORR and OER. The degree of activity depended on the transition metal (Co, Ni, and Mn) that was inserted into the octahedral sites of the spinel lattice [112]. Zhao et al. synthesized FeCoNC with two distinct structures, including star-like and dodecahedron-like structures. The star-like catalysts had a greater specific surface area than the dodecahedron-like catalysts due to their intricate structure. The unique structure allowed the star-like catalysts to expose more active sites, thus enhancing their catalytic activity for both ORR and OER (Fig. 6a, b) [113].

Due to the unique coordination environment and properties of different facets, specific facets with low activation barriers might be exposed to achieve maximum activity toward oxygen reactions [114, 115]. The (112) high-index facet has a more open structure with higher stepped atom density and density of catalytic active Co^3+^ sites compared to the (001) and (111) facets. Han et al. synthesized Co_3_O_4_ nanocrystals enclosed by the (001), (001) + (111), and (112) planes and found that the Co^3+^_Oh_ species on the (112) plane acted as superior active sites in terms of adsorption, activation, and desorption, as determined by DFT calculations (Fig. 6c–e) [116]. In another study, Xie et al. fabricated Ni–Fe alloy nanoparticles with (111) and (200) facets and found that the Zn-air batteries exhibited superior performance when assembled with (200)-faceted nanoparticles, due to the significantly high compressive strain exerted by these particles (Fig. 6f, g) [117].

While exposing specific facets can enhance the activity of electrocatalysts for ZABs, it can be challenging to maintain the crystal state of the catalysts throughout the electrochemical test. This is because the preferential orientation of the facet in catalysts often leads to the presence of grain boundaries, defects, and other factors that can also strongly influence the performance of ZABs [118]. Additionally, catalysts are sensitive to the reaction condition and might undergo irreversible reconstruction during preparation and reaction. In situ characterization can help distinguish the evolution of crystal structure and facets; In situ techniques are discussed later as a new concept.

#### Amorphization

Amorphization in an electrocatalyst increases the number of unsaturated coordination sites, which can improve the catalytic activity more effectively than the crystalline form [119, 120]. However, amorphous materials generally have poor conductivity and need to be loaded, deposited, or grown on conductive supports like carbon materials to facilitate electron transport and enhance electrical conductivity, which can result in lower ORR/OER overpotentials [121, 122].

Xu et al. reported the synthesis of Cu nanocluster/FeN_4_ amorphous composites embedded in N-doped graphene (Cu/Fe–NG), which has an amorphous structure that provides abundant and accessible active sites critical for the boosted ORR activity [82]. Amorphous oxides based on Fe, Co, or Ni with unparalleled 3d orbits showed excellent ORR performance, compared to the ORR performance of their corresponding crystalline counterparts. Carbon materials also have abundant active sites for the nucleation of oxides and avoid agglomeration. Liu et al. prepared a NiFePO–C composite as a cathode catalyst for ZABs, which showed stable cycling for more than 100 h [123]. Amorphous catalysts generally possess a large number of vacancies formed by lattice defects and disordered structures, which provide isotropic ion diffusion pathways and superior electrocatalytic performance [124]. Specifically, layered CoO (L–CoO) with long-range disorder structures have abundant ample oxygen vacancies and abundant oxygen chemisorption sites; these greatly enhance the ORR and OER performance and improve electrical conductivity [125].

Several studies used metal carbides (WC [126], TiC [127], Mo_2_C [128], B_4_C [129] etc.) and metal borates (M–B) as substrates as they exhibited excellent ORR/OER performance, owing to the large negative charge and electronic adjustment in the electrochemical processes [130, 131]. For example, Zhao et al. synthesized an amorphous electrode with iron borate nanolattice on the surface of nickel foam, in which the borate anions modified the electronic structure of transition metals and accelerated oxygen reactions [132]. Based on their findings, Saad et al. embedded crystalline W_2_CoB_2_ into the amorphous CoOOH layer (Fig. 6h), which resulted in high OER performance [133].

In spite of the commonly reported progress in ORR/OER catalytic activity, the working principle of amorphous composites in boosting the ORR/OER is still unclear. The current working theory is based on a combination of the following: (1) the electronic structure of metal sites is tailored by amorphous composites, and the electrocatalytic reaction barrier can be modified; (2) the vacancies can act as reaction sites and enhance the reaction kinetics [134, 135]. In addition, amorphous materials suffer from poor chemical and structure stability, which hinders the practical applications in ZABs.

### Interface Strategy

Rechargeable ZABs rely on a gas–liquid-solid triple-phase electrochemical reaction, where the surface/interface of the electrocatalyst is essential for determining the density, electrical conductivity, and reaction energy barrier of the exposed active site [136, 137]. Thus, the surface/interface of the electrocatalyst influences the performance of rechargeable Zn-air batteries.

The interface can be divided into the metal–metal interface, the metal–metal compound interface with the same or different metal, and the catalyst-substrate interface. The Co/CoFe heterointerface developed by Niu et al. is an excellent illustration of how metal–metal interfaces can enrich catalytic active sites and promote charge transfer between different components, which in turn can enhance electrocatalytic performance [138].

**Fig. 7:**
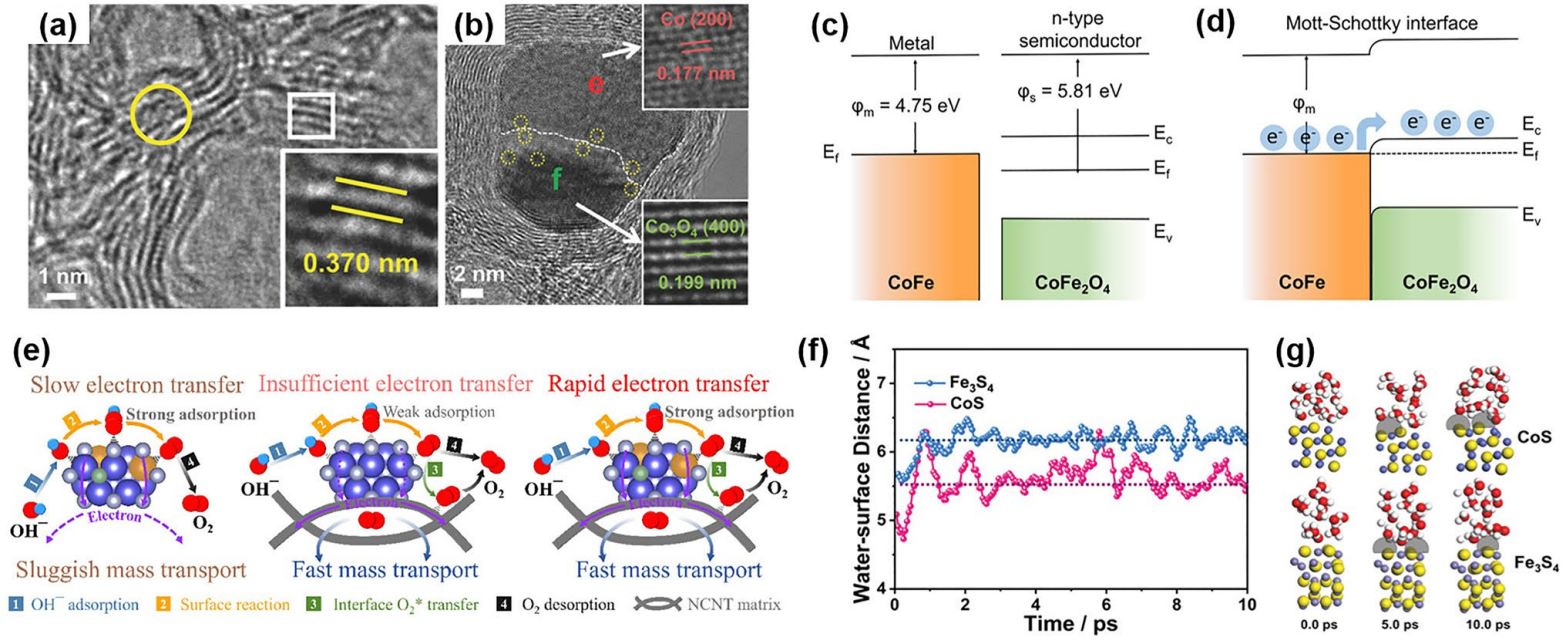
**a**, **b** HRTEM images of the interface between Co and Co_3_O_4_. Reprinted with permission from Ref. [139]. Energy band of CoFe alloy and CoFe_2_O_4_ **c** before and **d** after Mott-Schottky contact. Reprinted with permission from Ref. [140]. **e** Schematic illustration of electron and mass transport during the OER process on FC–Ni_3_N (left), Ni_3_N/NCNT (middle) and FC–Ni_3_N/NCNT (right). Reprinted with permission from Ref. [144]. **f** AIMD simulations of water layers on CoS and Fe_3_S_4_. Reprinted with permission from Ref. [146]

The metal–metal compound interface at the atomic scale can greatly enhance catalytic performance due to a synergistic effect. Many researchers have investigated interfacial interactions for the ORR/OER. Jiang et al. synthesized a hybrid material composed of interpenetrating metallic Co and spinel Co_3_O_4_ "Janus" nanoparticles. The interfaces between the two components served as atomic traps that imparted exceptional adsorption capability for adsorbing oxygen reactants (Fig. 7a, b) [139]. Baeck et al. prepared well-defined heterointer-faces between CoFe alloy and spinel-type CoFe_2_O_4_. The DFT calculations showed that the development of strongly coupled heterointerfaces between the CoFe alloy and n-type bimetal oxide (CoFe_2_O_4_) can generate a Mott-Schottky barrier, which can induce band structure bending and facilitate spontaneous electron transfer from the CoFe alloy (which has a higher work function) to CoFe_2_O_4_ (which has a lower work function) (Fig. 7c, d), thus enhancing the catalytic activity [140]. Wu et al. found that a heterogeneous interface of multicomponent cobalt oxides can generate rich oxygen vacancies as active sites, which can combine with the mixed valence of Co^2+^ and Co^3+^ to produce a Co/CoO/Co_3_O_4_/NCS catalyst with high ORR/OER performance [141]. Transition metal phosphides were found to be a promising class of oxygen electrocatalysts. Niu et al. developed a hetero-structural hybrid with a closely integrated CoO/Co_x_P interface that increased the accessibility of active species and facilitated smooth electron/reactant transportation. Because of these features, the newly-developed electrocatalyst outperformed the state-of-the-art non-noble-metal catalysts and even noble-metal benchmarks [142]. Guo et al. developed a Cu_3_P/TiO_2_ interface to optimize the adsorption of oxygen intermediates, shorten the electronic/mass transport path, and enhance the kinetics of the ORR reaction [143].

In electrocatalysis, the interface between the catalyst and the substrate is also significant. For example, Lu et al. anchored Ni_3_N onto the surface of NCNT to create a heterointerface, which increased the transport of electrons and oxygen between Ni_3_N and the conductive NCNT matrix (Fig. 7e) [144]. Similarly, Li et al. embedded Co nanoparticles in N-doped carbonized wood (Co@NCW). The interface between Co nanoparticles and N-doped graphitic carbon shells provided a fast electron transfer pathway at the interface and produced a synergistic effect that enhanced the electrocatalytic performance [145]. Yan et al. developed a hydrophobic-aerophilic strategy to fabricate CoS/Fe_3_S_4_ nanoparticles encapsulated in S, N co-doped carbon plate arrays. They found that the hydrophobic-aerophilic surface of the S, N co-doped carbon plate arrays created a favorable environment for the CoS/Fe_3_S_4_ nanoparticles to exhibit excellent OER activity. The ab initio molecular dynamics (AIMD) simulations showed that the hydrophobic surface repelled water molecules, creating abundant solid–liquid–gas three-phase reaction interfaces, while the aerophilic surface exposed Fe-sites and promoted the diffusion of reactive molecules/ions across the interface and the adsorption of oxygen (Fig. 7f) [146].

Interfacial catalysis is extensively applied in ZABs and shows satisfactory performance [147]. Although many studies have investigated the role of interfaces in electrocatalysis, there is still no consensus among researchers on the actual active center. Hence, whether the active center is the interface, the compounds formed at the interface, or the disorder/vacancies created by the interface is unclear. Further studies should be performed to better understand the underlying mechanisms of electrocatalysis at the interface. Researchers also need to assess the controllable interface and quantify the density of the interface to optimize the performance of ORR and OER.

### Atomic Engineering

#### Vacancy

**Fig. 8:**
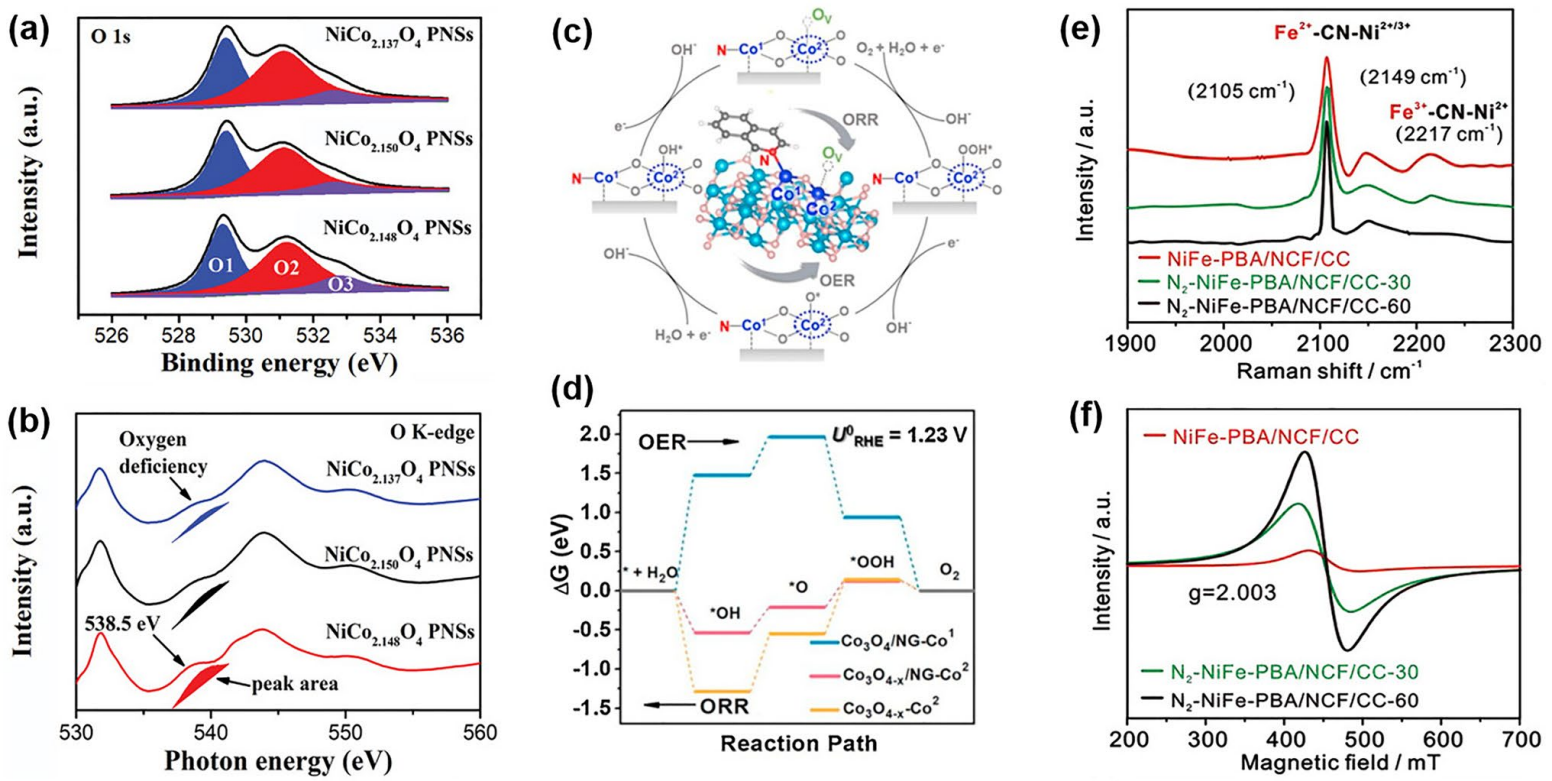
**a** O 1*s* spectra and **b** O K-edge XANES spectra for NiCo_2.148_O_4_, NiCo_2.150_O_4_, and NiCo_2.137_O_4_ PNSs. Reprinted with permission from Ref. [151]. **c** ORR and OER mechanisms over Co_3_O_4_-x/NG. **d** The calculated free energy diagram of ORR/OER for three different Co sites. Reprinted with permission from Ref. [152]. **e** Raman and **f** ESR spectra of different NiFe samples. Reprinted with permission from Ref. [154]

Vacancy engineering is performed to enhance catalytic activity by removing atoms from the bulk catalysts, which can modify the electronic structure of adjacent atoms [148, 149]. The oxygen vacancy (*V*_O_) is the most common type of deficiency, which can serve as an active site in oxygen electrocatalysis and enhance electrical conductivity and the adsorption of reactants [150]. To understand the relationship between *V*_O_ and ORR/OER performance, Tian et al. prepared various NiCo_2_O_4_-based mesoporous nanosheets (PNSs) with different levels of *V*_O_ by adjusting the calcining time. The concentration of *V*_O_ can be determined by performing X-ray photoelectron spectroscopy (XPS) and extended X-ray absorption fine structure spectroscopy (EXAFS) (Fig. 8a, b) [151]. The DFT calculations indicated that the activation energy of the rate-limiting step of ORR and OER was lowered by abundant *V*_O_. The results of the electrochemical tests showed that appropriate oxygen vacancies can promote electrochemical activity, but excessive oxygen vacancies can cause morphological changes and structural collapse, resulting in a decline in performance [151]. Heteroatom doping can enhance oxygen vacancies, as reported by Qin et al. Co single atoms on N-doped graphene can induce oxygen vacancy, which can regulate the charge density and optimize the electronic structure of Co sites. This can help in producing an optimal electrocatalyst with reduced overpotentials for both ORR and OER (Fig. 8c, d) [152].

Introducing other atom vacancies can also modulate the electrocatalytic performance of nanomaterials [153]. For example, Lai et al. reported an in situ CN vacancy developed by N_2_-plasma activation of NiFe-PBA catalysts. Raman and electron spin resonance (ESR) spectra were used to confirm the presence of *V*_CN_ (Fig. 8e, f) [154]. Song et al. synthesized nickel hydroxide with adjustable levels of nickel vacancies (*V*_Ni_) and showed that *V*_Ni_ significantly helped in the transformation of Ni(OH)_2_ into active NiOOH species [155].

**Fig. 9:**
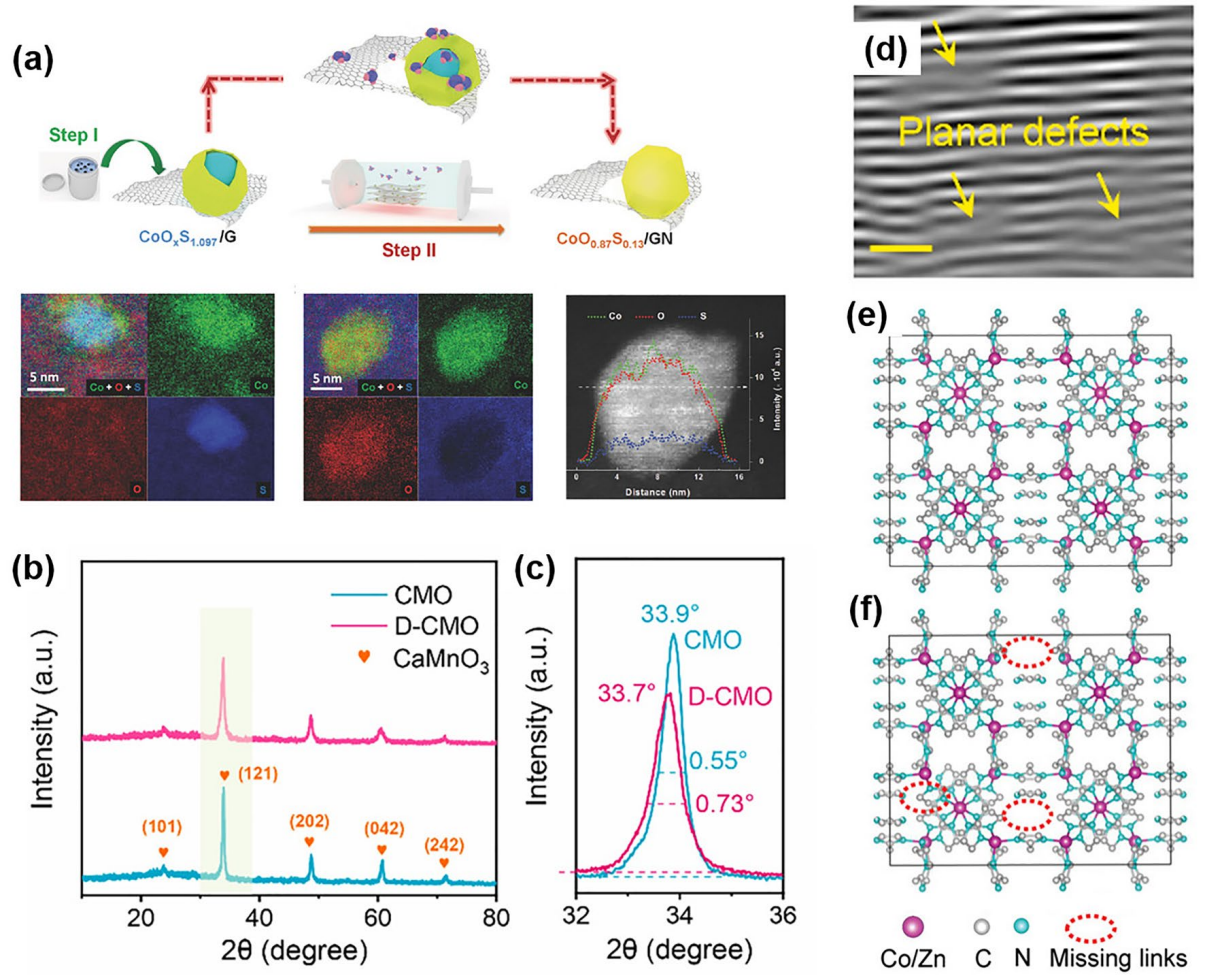
**a** Schematic illustration of the synthesis of CoO_0.87_S_0.13_/GN. Reprinted with permission from Ref. [158]. **b**, **c** XRD patterns, **d** FFT image of D-CMO. Reprinted with permission from Ref. [159]. Schematic diagram of the **e** intact ZIFs and **f** linker-deficient ZIFs. Reprinted with permission from Ref. [160]

Vacancy defects are also used to generate intrinsic vacancies and alter grain boundaries, which can provide feasibility to regulate their structure and enhance activity [156]. This is usually achieved via high-temperature treatment, chemical etching, or decreasing thickness. For example, Tao et al. produced edge-rich graphene by Ar-plasma etching and found that the edge-rich graphene had higher oxygen adsorption energy and more catalytically active sites for ORR than pristine graphene, determined by DFT calculations [157]. Fu et al. used the ammonolysis method and created CoO_0.87_S_0.13_ on graphene with an abundance of surface defects (Fig. 9a). The combination of oxygen-vacancy-rich, nonstoichiometric cobalt oxysulfides, and edge-nitrogen-rich graphene nanomeshes results in high electrocatalytic performance for ORR and OER [158]. Regarding high-temperature treatment, some phase transformations usually occur along with disordering of the crystal properties to some extent by controlling the calcination parameters. Yang et al. described a method to improve the activity of CaMnO_3_ (CMO) by ammonia heat treatment to perform the octahedral deformation of CMO (denoted as D-CMO, Fig. 9b–d) [159]. Some researchers performed theoretical calculations and found that the surface electron redistributions induced by octahedral distortion can lead to strong σ-type electronic coupling between the surface and adsorbents, which can enhance the ORR/OER activity of D-CMO, as the highest occupied molecular orbital (HOMO) and the lowest unoccupied molecular orbital (LUMO) are mainly distributed on this surface. Similarly, Yang et al. created linker defects in MOFs by removing some of the imidazole molecules from zeolitic imidazolate frameworks (ZIFs), which enhanced the activity of metal sites and electron transfer capability, resulting in outstanding bifunctional ORR and OER performance (Fig. 9e, f) [160]. It is essential to emphasize the intricate role of defects in electrocatalysis. Defects, while potentially contributing to beneficial effects, can also diminish activity. Additionally, they might function as recombination centers or undesired trap states.

#### Dopant

**Fig. 10:**
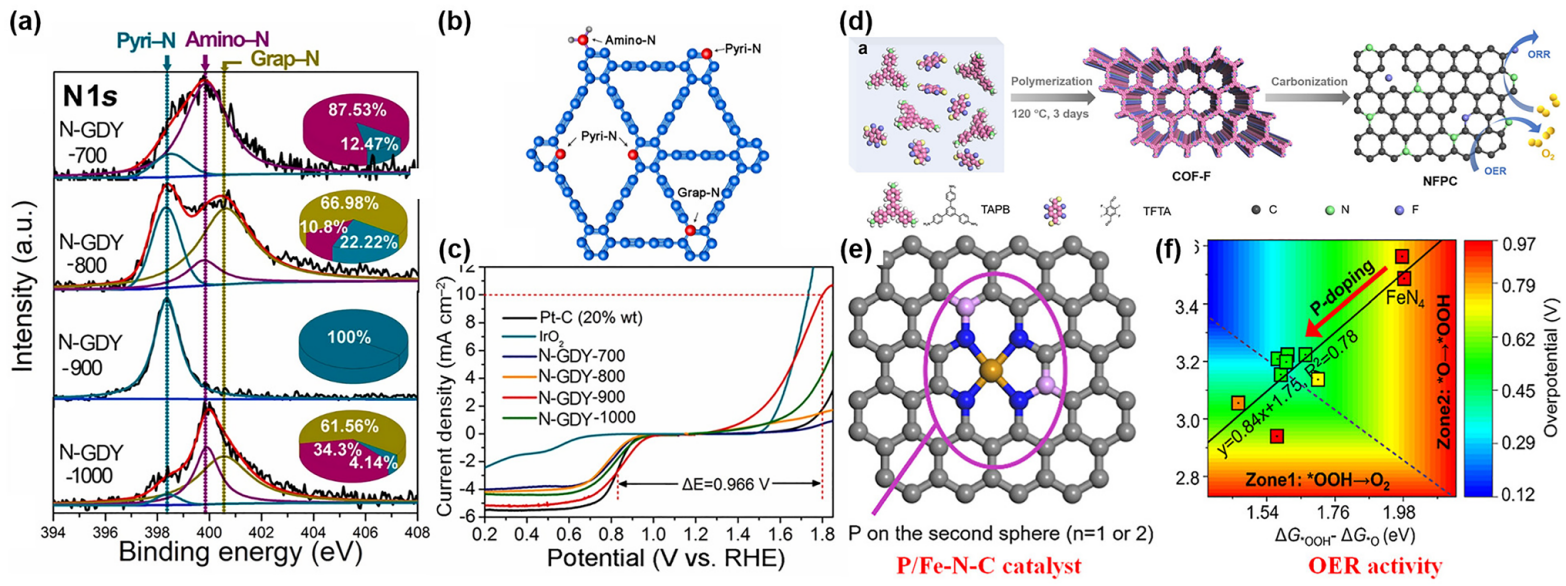
**a** XPS spectra of N1*s* for the samples after calcination in different temperatures. **b** Diagram of different configuration of N doping. **c** ORR and OER performance of four catalysts compared with commercial Pt/C and IrO_2_. Reprinted with permission from Ref. [174]. **d** Schematic illustration of the polymerization/crystallization of the COF-F and its subsequent carbonization for preparing NFPC. Reprinted with permission from Ref. [175]. **e** Schematic diagram of P/Fe–N–C catalyst. **f** Contour plot of OER overpotential as a function of Gibbs adsorption energies. Reprinted with permission from Ref. [179]

Many researchers have investigated and tested suitable doping elements for carbon substrates, as the electrically neutral carbon atoms of pristine carbon have poor activity toward the oxygen reactions [161, 162]. After the heteroatoms (N [163, 164], P [165, 166], B [167, 168], S [169, 170] etc*.*) doping, electrons are redistributed on nearby carbon atoms and act as the active sites for oxygen electrocatalysis. N-doping with various types of graphitic N, pyridinic N, pyrrolic N, and oxidized N has been extensively studied [171]. The type and concentration of N-doping in the carbon substrate are critical factors influencing the activity [172, 173]. For example, Lu et al. regulated pyridinic N (Pyri-N), graphitic N (Grap-N) and Amino- N on graphdiyne (GDY) via calcination temperature. With the concentration of pyri-N increasing, the ORR and OER performance have obvious advantages over other N-doped GDY catalysts, indicating pyri-N is the most favorable bonding mode for the oxygen reactions (Fig. 10a–c), being consistent with the results calculated by DFT [174]. Sun et al. prepared N, F co-doped porous carbon (NFPC) as bifunctional electrocatalysts for ZABs (Fig. 10d), where the DFT calculations showed a reduction in the depth of the local minima in the energy step diagrams, suggesting enhancement of OER and ORR [175].

The introduction of heteroatoms can sometimes regulate the local coordination environment [176, 177]. Meng et al. fabricated F, N-co-doped FeN_*x*_ moieties, which stabilized single Fe atoms and occupied vacancies during the defluorination process. This favored the adsorption of oxygen species and improved the performance of the oxygen reaction [178]. Zhou et al. investigated the effect of phosphorus on the electrocatalytic activity of FeN_4_-based catalysts. The FeN_4_ sites are the active sites for ORR, and their catalytic activity depends on the balance between the adsorption of *OOH and *O species. The researchers found that the addition of phosphorus effectively balanced the adsorption of *OOH/*O, which enhanced the ORR activity of the catalysts (Fig. 10e, f) [179].

Moreover, topological defects are often induced by heteroatom doping [180, 181]. Although carbon materials have high conductivity, their non-polar surface interacts weakly with oxygen-containing feedstocks, which results in poor electrocatalytic activity. To address this issue, topological defects, such as edge sites, lattice reconstruction, and dangling groups, can be introduced to regulate the electronic structure of carbon materials and create adsorption sites for oxygen-containing intermediates [182–184]. Yu et al. synthesized nitrogen-doped graphitic carbon nanosheets with numerous lattice defects that could change the catalytic properties of carbon materials [185].

Besides the carbon materials, a series of transition metal compounds including oxides [186, 187], sulfides [188, 189], phosphides [190, 191] and nitrides [192] have exhibited their potential for the catalysis of ORR and OER. However, conventional transition metal compounds without modulation show poor electrocatalytic activity [193]. Doping strategies can effectively manipulate the electronic structure and tune intrinsic ORR/OER electrocatalytic activity [194–196].

For example, in a Co-doped NiO electrocatalyst, electron transfer between Ni and Co enhanced the electrocatalytic activity [197]. Besides simple metal oxides, doping principles are also effective in complex oxides, such as perovskites, spinel, etc. Chang et al. investigated the effect of substituting Fe, N, and P tri-dopant in carbon nanotubes on the enhancement of the catalytic activity of ORR. The DFT results indicated that a suitable amount of P dopants could modulate the local electronic structure near the active sites of the catalyst, thus improving the ORR performance. The P dopants altered the electronic properties of the catalyst, creating new active sites for oxygen adsorption and reducing the activation energy required for ORR. The P dopants also enhanced the stability of the catalyst by reducing the formation of harmful intermediate species in the reaction [198]. Sulfides have drawn extensive attention because they can effectively enhance OER activity through simple methods. Lyu et al. proposed a continuous heat-treatment strategy to synthesize N and S-modified Co_9_S_8_ as electrocatalysts. The modified Co_9_S_8_ catalysts showed considerably higher ORR and OER activity than the non-doped catalysts. This enhancement was attributed to the introduction of N and S, which led to an increase in the defect concentration, adjustment of the Fermi energy level, improvement of electronic conductivity, and optimization of the energy barrier for oxygen adsorption reactions [199].

#### Atomic Dispersion

**Fig. 11:**
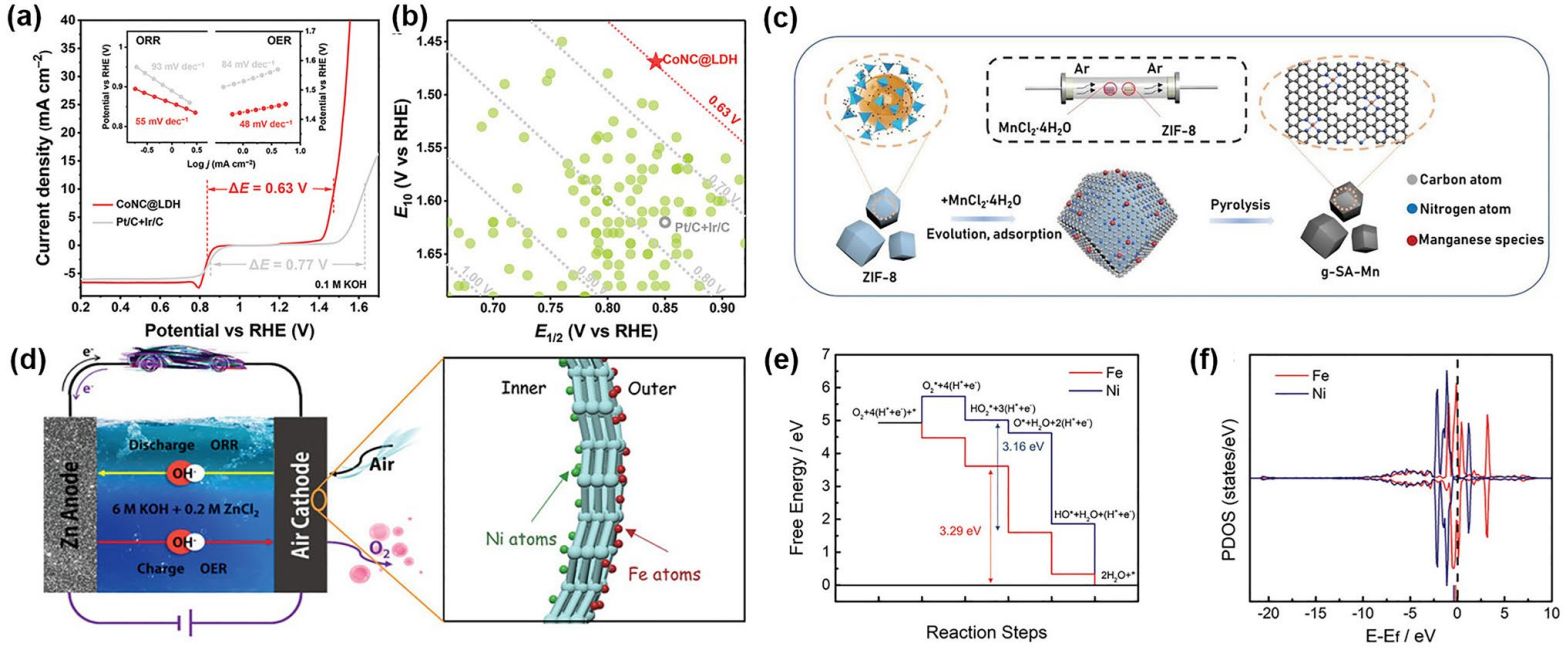
**a** LSV curves and Tafel plots of the CoNC@LDH and Pt/C + Ir/C for ORR and OER, respectively. **b** Descriptor of ORR and OER activity among CoNC@LDH and other reported bifunctional electrocatalysts. Reprinted with permission from Ref. [209]. **c** Schematic illustration of the preparation of atomically dispersed Mn catalysts. Reprinted with permission from Ref. [212]. **d** A schematic diagram of a Zn–air battery assembled with Ni–N_4_/GHSs/Fe–N_4_. **e** Free energies of OER and f PDOS of the single-atom sites of Fe–N_4_ and Ni–N_4_. Reprinted with permission from Ref. [214]

Since 2011, the concept of atomic dispersion has gained popularity in the field of electrocatalysis [200]. It can maximize the efficiency of transition metal atoms by facilitating precise regulation and rational design of intrinsic ORR/OER activity [201, 202]. The dispersed atoms, typically arranged in an M–N–C structure, where M represents a transition metal such as Fe, Co, and Ni, significantly improve the electrocatalytic activity even when present in small amounts [42, 203]. Some studies found excellent ORR catalytic performance of Co–N–C electrocatalysts [204–206]. Li et al. combined theoretical calculations and empirical findings to show that CoN_3_ sites were more favorable for the four-electron transfer in the ORR process than CoN_4_ sites [207]. Song et al. recently proposed a novel strategy to improve the performance of electrocatalysts by utilizing the synergistic effect between Co–N_2_ and Co–N_4_ moieties. The fully *π*-conjugated frameworks resulted from the presence of Co–N_2_ and Co–N_4_ moieties, which created a synergistic effect that enhanced the electronic conductivity and catalytic activity of the material [208]. Integrating multiple components in the composition of electrocatalysts allows for the selection of active sites for distinct mechanisms of ORR and OER, resulting in a generally superior bifunctional activity. Zhao et al. adopted this approach by selecting Co–N–C species and NiFe LDHs as ORR and OER active sites and integrating them into a composite (CoNC@LDH) for ZABs, which exhibited remarkable activity for both ORR and OER (Fig. 11a, b) [209].

Besides the Co–N–C active sites, M–N–C catalysts with different transition metal atoms at the center also exhibit acceptable catalytic activity [210]. Zong et al. synthesized M–N–C catalysts with different metal atoms at the center (M: Cu, Fe, Co, and Ni) on porous graphitic carbon nanospheres and found that Cu-SAs@N-CNS exhibited the highest ORR activity among the tested catalysts. The Cu–N–C catalysts showed exceptional electrocatalytic activity for both ORR and OER, making them highly efficient air cathode materials for ZABs [211]. Zhou et al. developed highly effective ORR catalysts in the form of atomically dispersed Mn-doped carbon materials (g-SA-Mn). The support for trapping and anchoring Mn-containing gaseous species was provided by porous zeolitic imidazolate frameworks. Through a high-temperature pyrolysis process, atomically dispersed Mn–N_*x*_ active sites were generated, which greatly enhanced the ORR catalytic performance of the material (Fig. 11c) [212].

The activity of pristine M–N–C sites can be enhanced by introducing a foreign metal site to create dual-atom catalysts [213]. Chen et al. placed nickel (Ni) and iron (Fe) single atoms on the inner and outer walls of graphene hollow nanospheres. The DFT calculations showed that the outer Fe–N_4_ clusters significantly enhanced the ORR activity, while the inner Ni–N_4_ clusters enhanced the OER activity (Fig. 11d–f) [214]. Wang et al. proposed a novel approach to optimize the electronic hybridization in Fe-based catalysts by introducing Se atoms. They also used DFT calculations to elucidate the underlying mechanisms of the observed phenomenon. The calculations suggested that the Se-doped Fe-based catalysts interacted strongly with the intermediates, which facilitated the adsorption and desorption of the intermediates during the catalytic process [215].

To summarize, the atomic dispersion structure has several advantages over other types of electrocatalysts, such as low metal dosage, high metal utilization, and uniform active sites. However, it has certain limitations, such as agglomeration and loss of single atoms during preparation and operation, which can lead to relatively poor stability.

## Mechanistic Understanding of Bifunctional Electrocatalysts

Heterogeneous catalysis is the most common reaction in ORR and OER, in which the activity is accelerated by the used catalysts. It is important to develop a proficient catalyst and further for practical application. The improvement of computing power and advanced techniques has facilitated intelligent and efficient exploration of the behavior of electrocatalysts. In this section, we described several typical computation descriptors and characterization techniques for catalysts. Through theoretical calculation and advanced characterization methods, the mechanism of bifunctional catalyst in ZABs was further analyzed.

### Theory Simulation

The performance of electrocatalysts in enhancing ORR and OER can be analyzed through first-principle calculations based on DFT and spin-polarized computations, which can be performed using the Vienna ab initio simulation package (VASP).

**Fig. 12:**
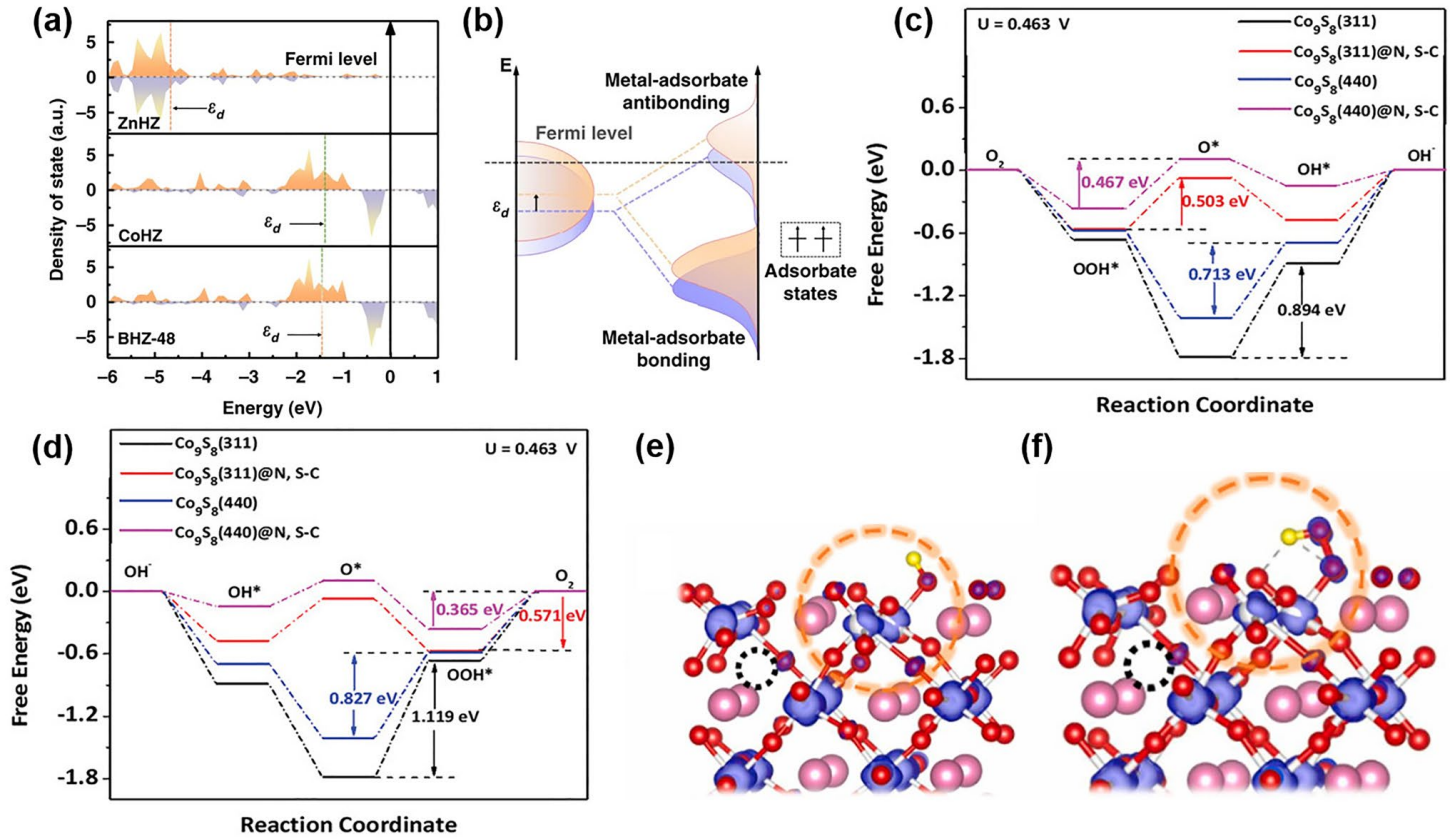
**a** The calculated d band density of state for the transition metal atoms in ZnHZ, CoHZ, and BHZ-48. **b** Schematic illustration explaining change of metal adsorbate interaction by altering the metal d band center (*ε*_d_). Reprinted with permission from Ref. [24]. Free energy diagram of various Co_9_S_8_ for **c** ORR and **d** OER pathways. Reprinted with permission from Ref. [199]. The HOMO of D-CMO (121) with **e** OH* absorption and **f** OOH* absorption, respectively. Reprinted with permission from Ref. [159]. FEM multiphysics modeling of O_2_ diffusion and O_2_ concentration contour near **g** the CoS/Fe_3_S_4_@SNCP and **h** CoS@SNCP air cathodes. **i** Three representative structures during AIMD simulations. Reprinted with permission from Ref. [146]

The density of states (DOS) of the catalysts can be used to detect the conduction band, valence band, and band gaps, as well as defects; thus, it can be used for improving the electrocatalytic ORR/OER activity. The d-band theory can be used to determine how the catalyst can improve the activity [216]. For example, as shown in Fig. 12a, b, the upshift of ε_d_ from CoHZ to ZnHZ reflected the partial charge transfer from Zn to Co, which decreased the energy level of Co d-orbitals [24]. The study used DFT calculations to determine the electronic properties of the MOFs and elucidate the charge transfer mechanism. Additionally, the energy level of the *p* orbitals relative to the Fermi level is an important factor in determining the adsorption capacity of oxygen-containing molecules [217]. A lower *E*_p_ relative to *E*_f_ indicates a higher ability of the catalyst to adsorb oxygen-containing species, which can enhance the electrocatalytic activity for reactions such as ORR and OER.

The ORR and OER processes generally involve the adsorption and activation of oxygen intermediates. The optimized adsorption configurations of reaction intermediates are crucial for enhancing the electrocatalytic performance of an electrocatalyst. For example, during the ORR, the adsorption of the OOH* and OH* intermediates on the surface of the catalyst are required to proceed through the four-electron pathway. In contrast, during OER, the adsorption of O* intermediate is necessary to proceed through the four-electron pathway [218]. Therefore, the optimized adsorption configurations of these intermediates can strongly influence the electrocatalytic activity of the catalyst. The theoretical calculations for the variation in free energy (∆*G*) related to the intermediates on active sites can be used to predict whether the reaction can proceed and the direction of the reaction. For example, Zheng et al. simulated the free energy diagrams of several models for ORR and OER. As shown in Fig. 12c, d, Co_9_S_8_(311)@N, S–C had the smallest barrier energy (0.503 eV) for ORR and the smallest barrier energy (0.571 eV) for the catalysis of OER among all the models, which explained its superior ORR and OER catalytic activity [199].

Orbital hybridization plays a key role in determining the reactivity and selectivity of electrocatalysts. Similar energy levels of the frontier orbitals of the catalyst and reactant facilitate the transfer of electrons, leading to efficient electrocatalysis. Yang et al. compared the lowest unoccupied orbital (LUMO) of CaMnO_3_ (CMO), and distorted CaMnO_3_ (D-CMO) and found that the oxygen has more orbital contribution for D-CMO. The O orbitals of the OH* and O_2_* adsorbates strongly interact with the surface of D-CMO, leading to a significant overlap and sharing of electrons. This σ-type electronic coupling indicates the formation of covalent bonds between the surface and the adsorbates, which can improve the catalytic activity of the material. The contribution of the O orbitals to the highest occupied orbital (HOMO) of the D-CMO further supports the presence of strong electronic coupling (Fig. 12e, f), which can improve the ORR and OER performance [159].

It should be noted that electrochemical systems are dynamic and exist in non-equilibrium states, which implies that theoretical simulations based on static surface models may not fully capture the complexity of the electrochemical reaction. Electrocatalysis of cathode catalysts occurs at the three-phase interface. A hydrophobic-aerophilic strategy has been devised to construct a self-supported air cathode utilizing CoS/Fe_3_S_4_ nanoparticles enclosed within S, N co-doped carbon plate arrays (CoS/Fe_3_S_4_@SNCP) to enhance the reaction kinetics at the air cathodes [146]. The finite element method (FEM) and ab initio molecular dynamics (AIMD) simulations were employed to delve deeper into the elucidation of the microenvironment origin for the catalysts. The FEM results indicate that the O_2_ concentration at the hydrophobic CoS/Fe_3_S_4_@SNCP electrode (Fig. 12g) surpasses that at the hydrophilic CoS@SNCP electrode (Fig. 12h), implying that the enhanced hydrophobic and aerophilic properties of the CoS/Fe_3_S_4_@SNCP electrode can effectively expedite the diffusion of oxygen at the triple-phase interface. Furthermore, in representative structures from AIMD at 5.0 and 10.0 ps, as depicted in Fig. 12i, the formation of hydrophobic cavities emphasizes the hydrophobic nature of the CoS and CoS/Fe_3_S_4_ surfaces (indicated by the black shadow). This underscores that the observed formation of hydrophobic cavities through AIMD simulations and this comprehensive simulation approach offers crucial information about the microscopic mechanisms and dynamic processes governing the behavior of the catalysts. To more accurately reflect the chemical reactions occurring in these systems, more precise characterizations are necessary.

### Data-Driven Machine Learning

**Fig. 13:**
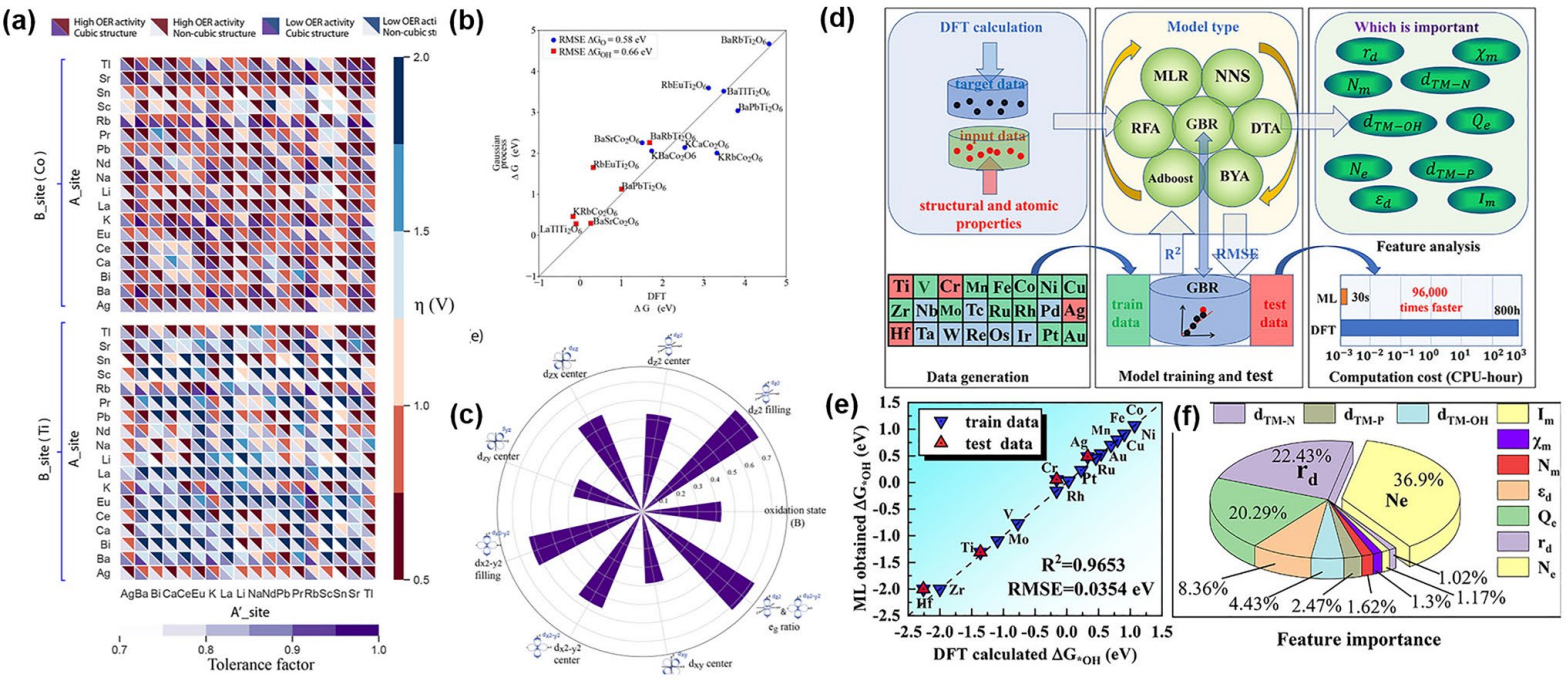
**a** Heat map visualization of the OER activity of double perovskites as a function of A-site/B-site cations in terms of the OER overpotentials and cubic phase probability. The red/blue color bar represent the overpotentials, and the purple bar represents the tolerance factor. **b** Parity plot for DFT-calculated vs. Gaussian process model prediction of descriptor adsorption free energies on candidate perovskite structures. **c** Polar distribution plots of the most informatic descriptors (KL entropy index > 0.4). Reprinted with permission from Ref. [219]. **d** Brief description of the ML process, divided into three parts, the data from DFT calculation, the ML model, and the feature importance analysis. **e** Calculated DFT ΔG *OH vs. ΔG *OH value obtained from ML by the GBR algorithm. **f** Feature importance of each descriptor with the corresponding proportion. Reprinted with permission from Ref. [220]

Active machine learning (ML) methods has many applications in a range of reactions, helping to accelerate research in areas such as material design, prediction of reaction mechanisms, and performance optimization. Thus, ML methods enable the handling of larger systems with greater accuracy for ORR and OER. Li et al. developed an adaptive ML strategy to search for high-performance ABO_3_-type cubic perovskites that can catalyze the OER. By learning the structure–reactivity relationships of approximately 250 initially computed perovskites, Li et al. developed an adaptive machine-learning strategy to analyze a larger set of double perovskites, including approximately 4000 AA’B_2_O_6_ double perovskites. Through this analysis, they identified about 10 stable cubic double perovskites with high OER catalytic activity. To identify these catalysts, they examined the feature distributions of the perovskites that were better or worse catalysts than LaCoO_3_. Their findings suggested that the electronic structure of the metal B-site plays a key role in determining the OER activity of perovskite catalysts. Using this information, the design of new and more efficient catalysts can be determined. Data-driven models can be used to easily identify these underlying factors and gain a better understanding of the structure–activity relationships of these materials. This highlights the potential of machine learning approaches for accelerating the development of new materials and catalysts (Fig. 13a–c) [219]. To design highly efficient bifunctional electrocatalysts for ORR and OER, Liu et al. conducted a study and found that V_Al_-2N_P_-AlP (where two nitrogen atoms replace two P atoms with an Al vacancy) is the most optimal choice for anchoring metal atoms. The DFT results indicated that the Co@V_Al_-2N_P_-AlP and Ni@V_Al_-2N_P_-AlP systems exhibit a low overpotential for OER and ORR, making them promising candidates for application in ZABs. The ML method used in that study also showed that the number of TM-d electrons (*N*_e_), the radius of the TM atom (*r*_d_), and the charge transfer of TM atoms (*Q*_e_) were the primary descriptors related to the adsorption behavior (Fig. 13d–f) [220].

The machine-learning method with large-scale simulations and massive experimental data is a new paradigm for discovering ideal catalysts, making it possible to conduct high-throughput science.

### Surface-Detection Techniques

While analyzing the electrochemical behavior of electrocatalysts in ZABs, their raw physiochemical properties and phase transformation in battery operation, as well as the corresponding changes in chemical composition and electrochemical performance, need to be determined. For this, advanced surface-detection techniques might provide useful information about electrified interfaces under operating conditions.

**Fig. 14:**
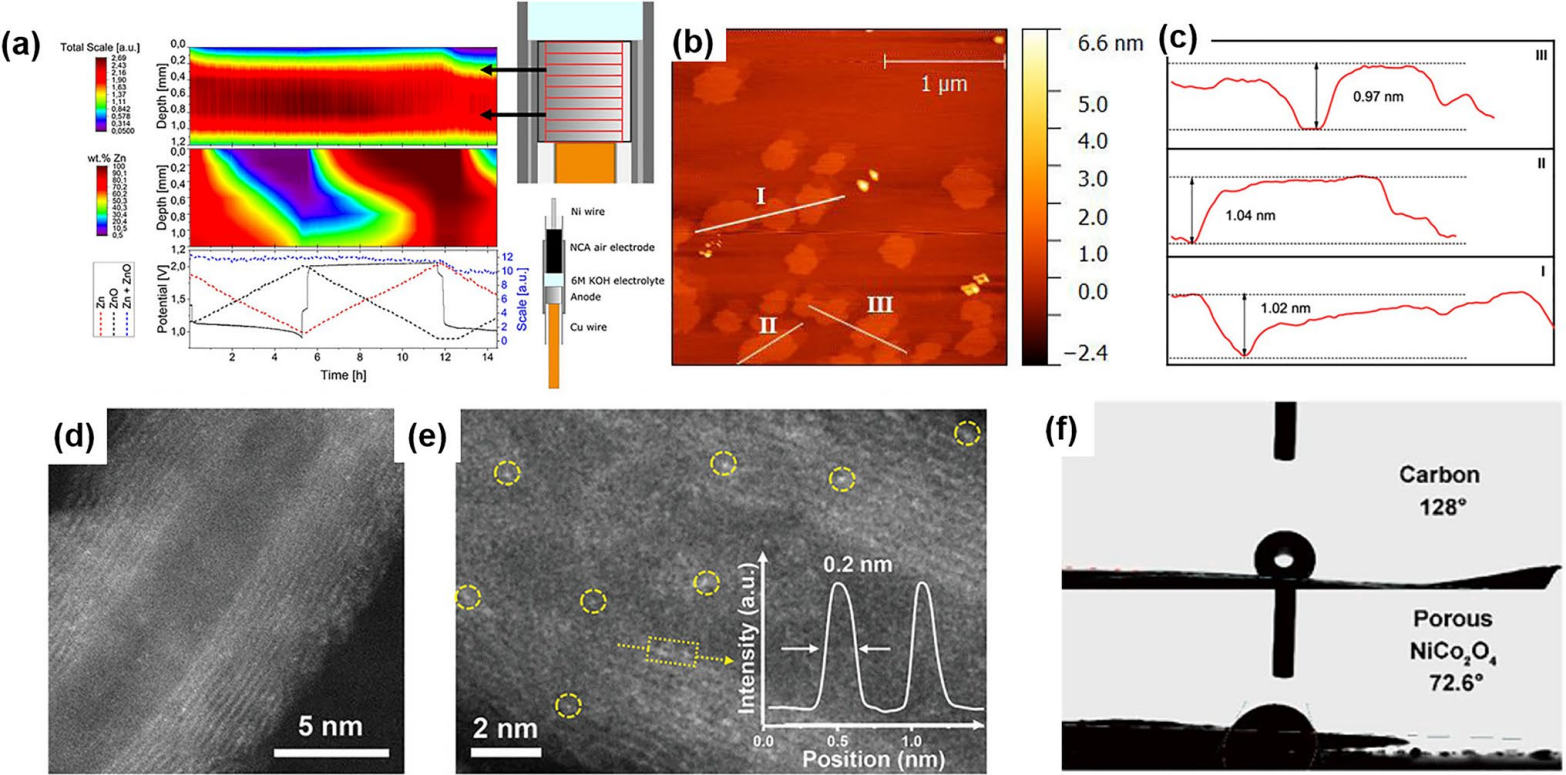
**a** (Top) A heatmap of the summed scale factors for Zn and ZnO. (Middle) wt% Zn heatmap showing the reaction front of the Zn/ZnO phase transformation. (Bottom) voltage profile over time, shown with the corrected scale factor for Zn and ZnO. (Right) A close-up of the anode subdivided in 0.2 mm × 1 mm segments and schematic diagram showing the capillary-based ZAB. Reprinted with permission from Ref. [221]. **b** AFM image and **c** the corresponding height profiles of Cu/Fe-NG. Reprinted with permission from Ref. [82]. **d**, **e** HAADF-STEM image and corresponding EDS mapping images on N, Co, Ni, Fe, and O elements of CoNC@LDH. Reprinted with permission from Ref. [209]. **f** Comparative testing of the contact angles at air–water-solid catalyst interfaces. Reprinted with permission from Ref. [222]

The commonly used methods for surface characterization include X-ray diffraction (XRD), XPS, scanning electron microscopy (SEM), and atomic force microscopy (AFM), among other techniques. XRD uses X-ray diffraction patterns to obtain information about the composition of materials, as well as the structure and morphology of atoms or molecules within the materials. Graae et al. used in-situ XRD to analyze the phase transition of the anode between pure Zn and ZnO (Fig. 14a). Their results indicated that the effectiveness of the air catalyst to run the reverse reaction was not the limiting factor for an effective secondary Zn-air cell; instead, the main challenge was the morphological changes in the anode over time, which limited the access to ZnO nucleation sites near the surface, thus increasing the overpotential required to initiate the discharge reaction [221]. XPS can be used to determine the elemental and chemical composition of surfaces and thin films. It can also provide semi-quantitative information on the elemental composition of the sample surface by measuring the peak intensity of the XPS peaks. However, XPS is a surface-sensitive technique and can only provide information about the top few nanometers of the sample surface.

Scanning probe microscope (SPM) is a new microscope tool with several advantages over other types of microscopes and analytical instruments. SPM can provide high-resolution data. It can readily “see” atoms in a way that cannot be achieved via ordinary electron microscopes. In recent studies, many atom-thick nanosheets were characterized by SPM for analyzing their surface structure and physical thickness. For example, Li et al. studied exfoliation-derived metallic CuCo_2_S_4_ nanosheets with an atom-thick layer using an atomic force microscope (AFM; one type of SPM). The height of CuCo_2_S_4_ nanosheets was found to be 0.90–1.25 nm, reflecting a thickness of 4–6 atomic layers [189]. The results suggested that the ultrathin nanosheet structure of CuCo_2_S_4_ NSs provided exposed active sites, resulting in excellent electrocatalytic performance. Xu et al. reported the synthesis of Cu nanoclusters/FeN_4_ amorphous composites embedded in N-doped graphene (Cu/Fe–NG). They used AFM to observe its surface texture and thickness. In a 2 µm × 3 µm field of view, homogeneous contrast verified the uniform smooth surface of graphene. The corresponding height profiles revealed that Cu/Fe-NG had a thickness of 0.35–1.05 nm, indicating the presence of 1–3 stacked layers (Fig. 14b, c) [82]. High electrocatalytic performance was associated with the benefits of 2D nanostructures with atomic thickness, which increased the surficial active site ratio, improved the contact interfacial area, and decreased surface energy.

The dynamic interplay of the ORR and OER at triple-phase boundaries underscores the importance of optimizing surface hydrophilic/aerophilic properties, particularly within a static environment. Recognizing this critical aspect, an investigation into the contact angles on different catalysts was undertaken using a high-speed camera, as illustrated in Fig. 14f [222]. Notably, the porous NiCo_2_O_4_ catalyst exhibited a water–solid-air interface angle of 72.6°, in stark contrast to the 128° observed on the carbon surface. This distinction emphasizes the desirable hydrophilic nature of porous NiCo_2_O_4_, strategically balancing the contracting forces between gases and aqueous electrolyte.

### Atomic Scale Characterization

Atomic scale characterization methods can be used to elucidate the structure and properties of materials at the atomic level. Various methods can be used to characterize the atomic structure of materials, including Scanning Tunneling Microscopy (STM), Transmission Electron Microscopy (TEM), Raman, X-ray absorption spectroscopy, etc.

Aberration-corrected scanning transmission electron microscopy (AC-STEM) can be used to obtain atomic-resolution images at a low-order zone axis orientation. AC-STEM can distinguish the spatial atomic distribution and directly show the distribution and defective properties, as well as, disordered local symmetry. Zhao et al. used high-angle annular dark field scanning transmission electron microscopy (HAADF-STEM; a type of AC-STEM) to visualize single-atom Co inside the LDH, where the bright dots were resolved as Co atoms (Fig. 14d, e) [209]. Meng et al. reported another example regarding the spatial isolation of Fe species at the atomic scale by regulating the content of Co nanoparticles in MOF-derived carbon. This allowed for the preparation of nanoparticles, atomic clusters, and single-atom Fe catalysts on N-doped porous carbon [90]. The prepared Co nanoparticles and highly dispersed Fe loaded on N-doped carbon can provide ZABs with excellent bifunctional activity and durability. More intriguingly, the vacancy defects and rearrangement can also be observed by TEM. However, AC-STEM has difficulty distinguishing between elements with similar atomic numbers. Additionally, the coordination species, such as metal–carbon and metal–metal bonds, cannot be identified by AC-STEM.

**Fig. 15:**
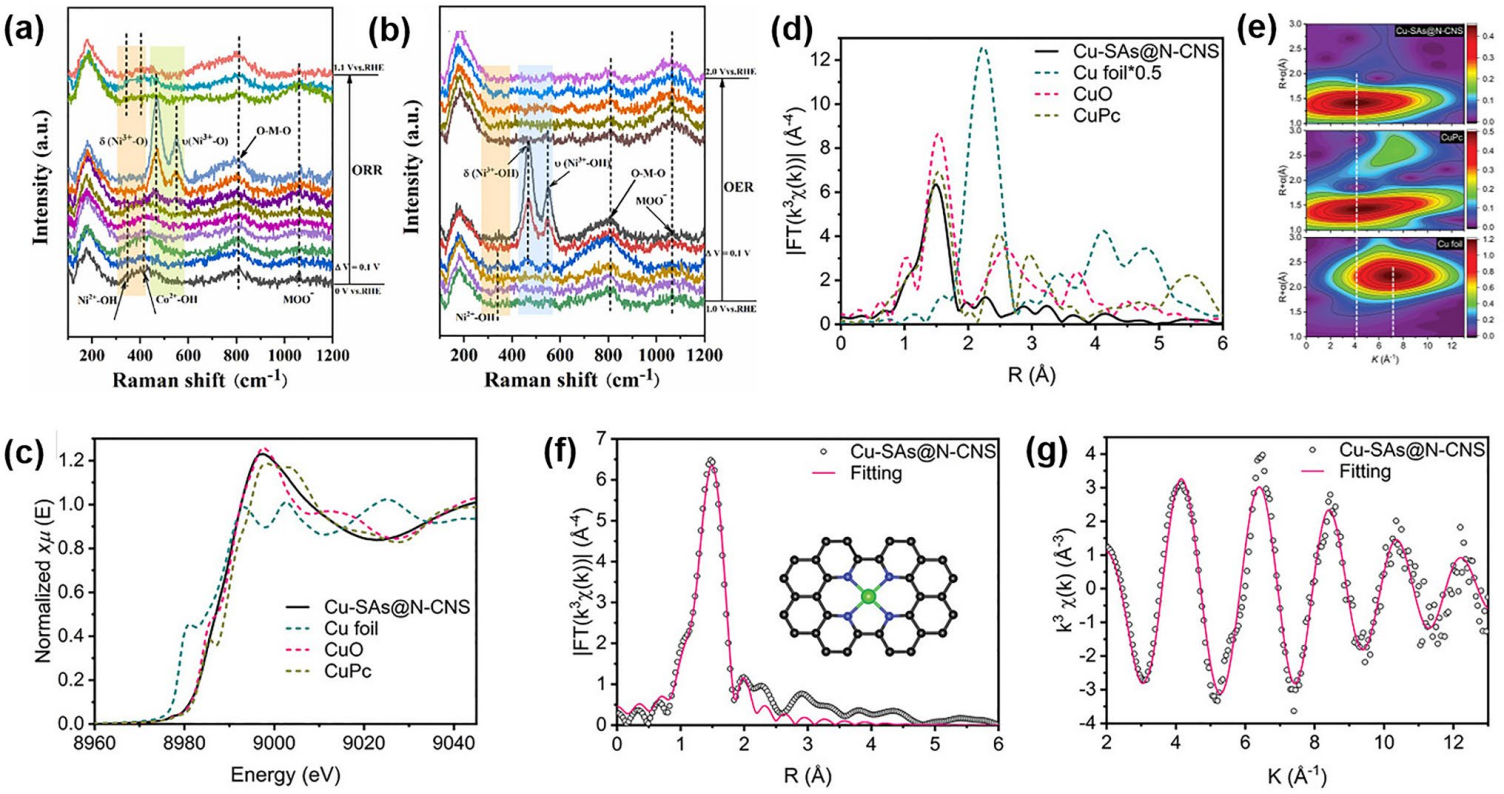
In situ Raman spectra collected on CoNi-CoN_4_-HPC-900 for **a** the ORR process and **b** the OER process in 0.1 M KOH solution. Reprinted with permission from Ref. [223]. **c** Cu K-edge XANES, **d** FT k^3^-weighted Cu K-edge EXAFS, and **e** WT-EXAFS spectra of Cu-SAs@N-CNS and Reference samples. **f**, **g** FT-EXAFS fitting curves for Cu-SAs@N-CNS in R and K spaces. Reprinted with permission from Ref. [211]

Raman spectroscopy is often combined with infrared testing as they complement each other. By assessing the Raman frequency, the substance can be identified, and its quality can be determined. Raman displacement changes correspond to material stress, tension, and doping, while peak strength can be semi-quantified for the substance. Changes in the peak width can indicate disorder and defects in the substance. Liu et al. used in situ Raman spectroscopy to elucidate the catalytic mechanism of CoNi catalysts for ORR and OER. During the ORR, the peaks showed the conversion of Co(III)/Ni(III) to Co(II)/Ni(II), while the OER process showed the opposite pattern, suggesting that the ORR and OER on CoNi catalysts occurred through the reduction and oxidation of M(II) and M(III) species (Fig. 15a, b) [223]. Furthermore, the electrocatalysts exhibiting microstructural changes and defects can be identified using Raman spectroscopy. This method allows for the real-time detection of alterations in chemical bonds.

Synchrotron-based X-ray absorption spectroscopy (XAS) is a new and powerful technique that can be used for characterizing the geometric structure of active sites in electrocatalysts. This technique can be divided into two energy regions, including the X-ray absorption near-edge structure (XANES) and the extended X-ray absorption fine-structure (EXAFS). XANES is highly sensitive to the oxidation states and the coordination structure, such as whether the metal species are in tetrahedral or octahedral sites. On the other hand, EXAFS is used to evaluate the local coordination and bonding environment of metal atoms. These two regions of XAS provide valuable information about the electronic and geometric structures of active sites in electrocatalysts [224]. For example, Zong et al. prepared Cu single atoms anchored to carbon (Cu-SAs@N-CNS). The valence state of Cu was found to be close to + 2 by XANES analysis, and the Fourier transformation of EXAFS results showed the existence of Cu-N_x_ coordination, where the x number was 4.1 (Fig. 15c–e). The Morlet wavelet transform (MWT) is a powerful technique for analyzing the local environment of metal complexes by directly visualizing multiple scattering signals from neighboring atoms in the EXAFS spectra. This technique can be used to effectively extract information about the geometric structure of metal complexes by analyzing the oscillations in the EXAFS spectra at different frequencies. The quantitative least-squares EXAFS curve-fitting analysis plot of Cu-SAs@N-CNS (Fig. 15f, g) showed a single contour peak with a maximum intensity at 4.2 Å^−1^, which was close to that of CuPc with Cu-N_4_ bonds. This suggested the presence of a Cu–N_*x*_ coordination configuration in Cu-SAs@N-CNS [211]. Zhang et al. introduced Mg into a three-dimensional ordered mesoporous material (denoted as 3DOM Mg_*x*_Co_3−*x*_O_4_). In the XANES spectra, the shift of 3DOM Mg_*x*_Co_3−*x*_O_4_ was consistent with that of MgO, indicating that the valence state of Mg was + 2. The contour plots of the EXAFS wavelet transforms for 3DOM Mg_*x*_Co_3−*x*_O_4_ and pristine Co_3_O_4_ confirmed the chemical coordination of Co–Co and Co–O, indicating that the inclusion of Mg in Co_3_O_4_ can modulate the electronic structure of 3DOM Mg_*x*_Co_3−*x*_O_4_ and enhance its intrinsic catalytic performance during charge and discharge processes [225]. Zheng et al. synthesized sulfur-doped NiCo-(oxy)hydroxysulfides. The results of the Morlet wavelet transform confirmed that the incorporation of S in the composites can effectively regulate the local environment and the electronic structure of the metallic sites, leading to enhanced catalytic activity and stability when used as anodes in Zn-air batteries [226].

Positron annihilation spectroscopy (PAS) is a technique used in materials science to study the properties of materials at the atomic and molecular levels. This method involves the interaction of positrons, which are positively charged antiparticles of electrons, with electrons in a material. PAS is widely acknowledged as a highly appropriate method for characterizing open volume defects [227]. The positron lifetime is subject to variation depending on the electron density surrounding the positron. Consequently, when positrons annihilate from defects, their lifetime is extended compared to that in defect-free materials. This distinction allows for the identification of potential defects through positron annihilation lifetime spectroscopy (PALS), wherein the extracted lifetime components serve as indicators of these defects [228]. Electron Spin Resonance (ESR), also known as Electron Paramagnetic Resonance (EPR), is a spectroscopic technique that investigates the interaction between unpaired electrons and an external magnetic field. In addition to PALS, electron spin resonance (ESR) can also be employed to characterize the degree of defects in materials.

### Characterization Under Operating Conditions

**Fig. 16:**
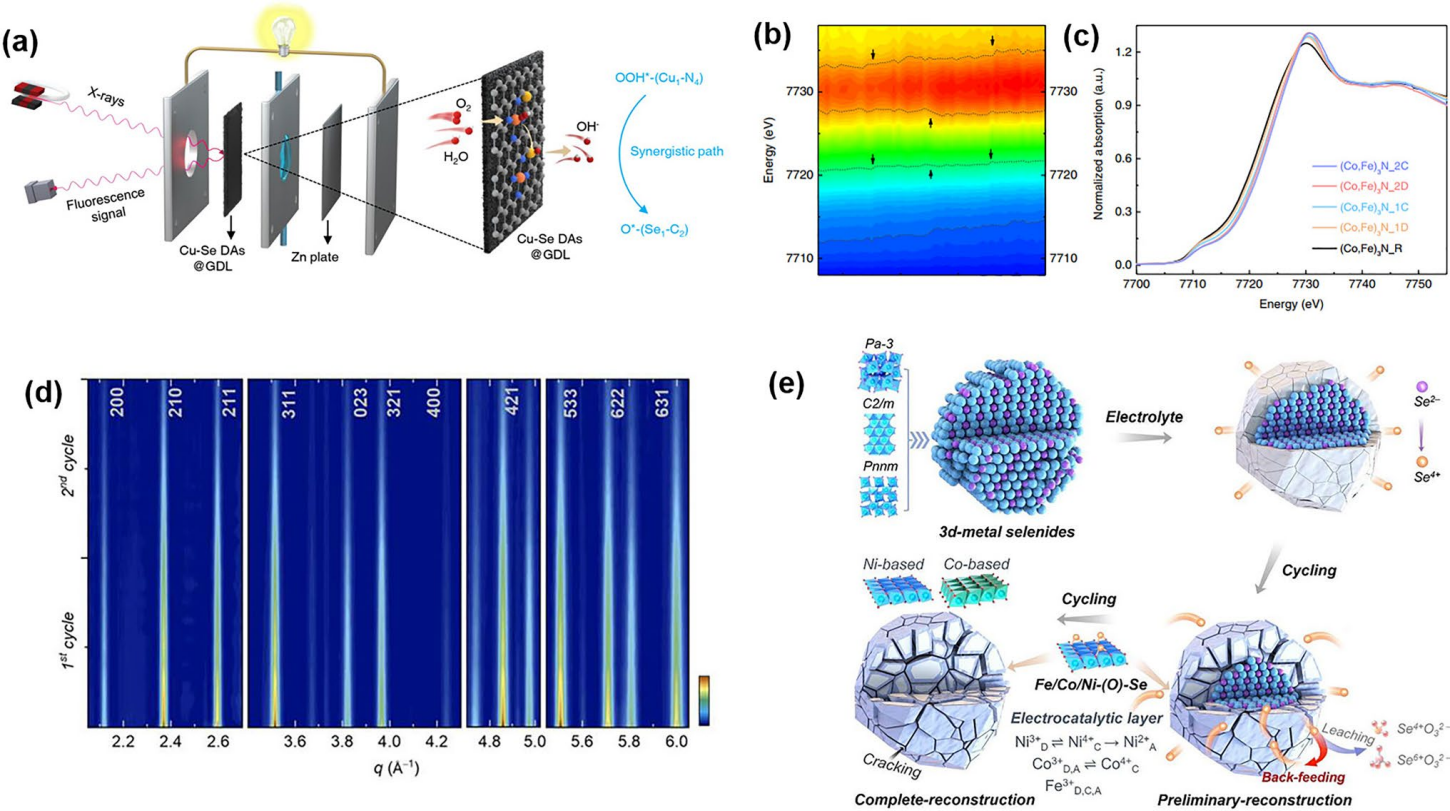
**a** Illustration of operando XAS set up for ZAB under realistic device working conditions. Reprinted with permission from Ref. [230]. **b**, **c** The operando XANES spectra of Co. Reprinted with permission from Ref. [192]. **d** Operando 2D HEXRD contour map of (Fe, Ni)Se_2_ in a Zn–air battery. **e** Schematic demonstration of the Se-driven reconstruction mechanism. Reprinted with permission from Ref. [231]

To explore the reversibility of the zinc–air battery and the specific role played by the bifunctional electrocatalysts, characterization is required to capture the structural and chemical transformation under battery-operating conditions, which is different from the common half-cell (namely three electrodes system: working electrode, reference electrode, counter electrode). The operational context is of utmost importance. Zheng et al. have innovatively incorporated thermoelectric devices and materials, seamlessly integrating the thermoelectric system with zinc–air batteries. This integration has resulted in a substantial improvement in the energy efficiency of ZABs [229]. The ZAB prototype with flowing electrolyte could eliminate electrolyte-induced performance variation to allow direct focus on the behavior of bifunctional electrocatalysts under operating conditions. Sun et al. designed an operando synchrotron radiation infrared spectroscopy (SRIR) device to investigate the realistic battery-operating conditions (Fig. 16a). The device facilitated the mass transport of O_2_ during ORR, which was different from the conventional half-reaction cell used in many studies [230]. Deng et al. investigate the evolution of Co species during the charge and discharge process in Zn-air batteries by in situ XRD and XAS. The Co K-edge XANES results showed that the valence state of Co continued to increase during the process, as shown in Fig. 16b, c. A dynamic illustration depicting the formation and development of a nanoscale oxyhydroxide shell is provided, with periodic shifts in the valence state of the performance-dominant element being observed [192]. The operando XAS and SRIR device provided valuable insights into the mechanism of Zn-air batteries by allowing for the in-depth investigation of the electrochemical and spectroscopic properties of the catalyst under realistic conditions. Recently, an electrolyte-induced layer-by-layer ZAB is employed to eliminate performance variation with operando HEXRD (Fig. 16d, e), suggesting the reconstructed phase during the stages of charge and discharge [231]. The procedure results in the formation of an amorphous (oxy)hydroxide featuring selenium motifs with shared oxygen bonds. This, in turn, initiates charge redistribution at metal sites, reducing the energetic barrier for their current-driven redox processes.

Researchers agree that oxygen electrocatalysis is a dynamic process. However, different electrocatalysts may experience different self-reconstruction processes during the cycling of ZABs, and it’s difficult to visualize accurately. In-situ characterizations, such as XRD, Raman, and XAS, provide accurate information on the surface structure, including changes in the local coordination environment, structure, and oxidation state of electrocatalysts during battery cycling. By using in situ detection techniques, distinct changes in the composition and structure of the electrocatalyst can be probed. During the reaction, operando detection can be further utilized to monitor the catalytic intermediates and microenvironments. The in situ techniques provide a reliable basis for conducting theoretical and experimental studies for screening catalytic systems.

## Summary and Outlook

High-performance zinc–air batteries represent a promising technology for next-generation rechargeable batteries, and efficient electrocatalysts are the key to advancing these batteries from the laboratory to the industry. In this review, we provided an overview of the mainstream methods used for fabricating non-noble-metal electrocatalysts for ORR/OER. Recent advancements in understanding catalyst engineering and elucidating the reaction mechanisms were made using theoretical calculations and advanced techniques. However, further investigation is necessary to perfect this field.

1. Although many high-performance ORR and OER catalysts have been developed over the past few years, researchers have failed to determine a universal structure–activity/selectivity relationship to guide catalyst design. The most frequently used strategies to develop an ideal catalyst involve optimizing the number of active sites and the intrinsic activity of each active site. Here, we outlined the principles of catalyst design in four major aspects, including morphology, crystal structure, interface, and atomic engineering. Most of these principles can be deduced from experimental results and computational simulations. Therefore, future studies need to determine the true structure-performance relationship and provide predictive guidelines for designing bifunctional catalysts for ORR and OER with optimized activity and durability.
2. Gas-solid-liquid triple-phase reactions at the cathode strongly influence the performance of ZABs. Identifying appropriate catalysts can help in the synergistic optimization of electron conduction, oxygen gas diffusion, and ion transport for electrocatalysis, which in turn can accelerate the understanding of the reaction mechanism at the triple-phase interface.
3. During battery operation, the performance of air-electrodes is degraded due to carbon corrosion/oxidation at oxidation potential in high-concentrated alkaline electrolyte. This inferior stability and cyclability greatly shorten the operation lifetime of ZABs. To minimize and further eliminate the employment of carbon might be one possible solution. Attempts to replace carbon with highly conductive metal-based substrates (such as carbides) have shown some interesting results. Although metal-based batteries have a satisfactory lifetime, their bifunctional activity cannot compete with that of carbon-based batteries, as carbon is also an excellent electrocatalyst besides the substrate. Therefore, highly active and conductive metal-based electrocatalysts need to be identified for long-lasting ZABs.
4. The evolution of most bifunctional electrocatalysts is still unclear due to the lack of theoretical prediction and in situ characterizations. To solve this problem, advanced operando characterization techniques, theoretical computations, and machine learning methods can be used together to achieve a fundamental understanding of the electrochemical behavior of electrocatalysts in ZABs. By using this strategy, vital information might be revealed that might be used as the design principles for further material development.
5. Most electrocatalysts are prepared in a highly controlled environment and at a laboratory scale, and thus, their application shows poor reproducibility at the industrial scale. Additionally, electrocatalysts with nanostructures are sensitive to electrochemical measurements, and their rechargeability cannot meet large-scale requirements. Thus, to meet the demands of industrial applications, inexpensive, easy-to-synthesize, and stable catalysts need to be developed.
